# New insights from museum specimens: a case of Viviparidae (Caenogastropoda: Mollusca) in Iwakawa’s collection preserved in the National Museum of Nature and Science, Tokyo

**DOI:** 10.3897/BDJ.8.e52233

**Published:** 2020-12-21

**Authors:** Takumi Saito, Osamu Kagawa

**Affiliations:** 1 Toho University, Narashino City, Chiba Pref., Japan Toho University Narashino City, Chiba Pref. Japan; 2 Tohoku University, Sendai City, Miyagi Pref., Japan Tohoku University Sendai City, Miyagi Pref. Japan

**Keywords:** Ancient lake, endangered species, extinct population, freshwater molluscs, taxonomic history

## Abstract

**Background:**

In this study, we clarify the classification of museum specimens of the family Viviparidae, which is composed of six species/subspecies in Japan, including three endangered species. We examined *Viviparus sclateri* specimens from the Tomotaro Iwakawa collection (1855-1933) in the National Museum of Nature and Science, Tokyo. The Iwakawa's collection was catalogued in 1919 and *Viviparus sclateri*, labelled with Naga-tanishi, the current Japanese name for *H. longispira*, which was, at the time, equivalent to *Viviparus sclateri*, was listed in this catalogue. The catalogue noted localities of *Viviparus sclateri* (Naga-tanishi) from outside Lake Biwa, including occurrences in Lake Kasumigaura and Lake Suwa. However, *Heterogen longispira* (Naga-tanishi) is currently considered to be endemic to Lake Biwa drainage. The actual status of *Viviparus sclateri* in Iwakawa (1919) has not been clarified until now.

**New information:**

Our examination revealed that *Viviparus sclateri* from Iwakawa’s catalogue included *H. japonica*, *H. longispira* and *Sinotaia quadrata histrica*, based on current taxonomy. Specimens assigned to *H. longispira* occurred only in Lake Biwa drainage. *Heterogen japonica* was confirmed to be present in all lots and some *H. japonica* from Lake Suwa had a distinctive morphology. *Sinotaia quadrata histrica* was only confirmed to occur in Lake Suwa. Furthermore, some specimens from southern Lake Biwa and the Seta River had intermediate characteristics between *H. japonica* and *H. longispira* and their populations are currently almost extinct.

## Introduction

Museum specimens provide valuable insights into the evolutionary and ecological history of living and extinct populations, their taxonomy and knowledge for conservation biology (Suarez and Tsutsui 2004). For example, morphological and molecular data on historical specimens assist in the clarification of the classification and various other biological issues (Wandeler et al. 2007). In this study, we focused on specimens of endangered freshwater molluscs in a collection of Tomotaro Iwakawa from the National Museum of Nature and Science, Tokyo. Tomotaro Iwakawa (1855-1933) was an early contributor to zoology in Japan and he researched freshwater molluscs in Japan with particular attention (Taki 1933). Furthermore, he established one of the earliest systematic malacological lists in Japan (Taki 1933) and specimens of the list were deposited at the National Museum.

Viviparidae Gray, 1847, belonging to Caenogastropoda, is a family of freshwater gastropods with 125–150 valid described species globally and have a wide geographic range in Asia (Hirano et al. 2019b, Van Bocxlaer and Strong 2019, Stelbrink et al. 2020). Historically, Japanese viviparid gastropods have been classified as four species, based primarily on conchological features (Habe 1990, Masuda and Uchiyama 2004): *Cipangopaludina chinensis laeta* (Martens, 1860), *Cipangopaludina japonica* (Martens, 1861), *Heterogen longispira* (Smith, 1886) and *Sinotaia quadrata histrica* (Gould, 1859). Recently, some attributions were modified by Hirano et al. (2019b), namely, *Cipangopaludina japonica* was modified to *Heterogen japonica*, based on molecular phylogeny. Furthermore, molecular analyses have led to the recognition of two subspecies of *C. chinensis* in Japan: *C. c. laeta* and *C. c. chinensis* and the existence of an undescribed species of *Heterogen* sp was also suggested (Hirano et al. 2019a, Hirano et al. 2019b). Thus, based on all the above-mentioned work on Japanese Viviparidae, we consider six viviparid species/subspecies to exist in Japan. Of these six species/subspecies, *H. longispira* is listed on the IUCN Red List as EN (Köhler and Rintelen 2011). Furthermore, *C. chinensis* is listed on the IUCN Red List as LC, although the subspecies is not designated (Köhler et al. 2012). These two species are also listed on the Red List issued by the Government of Japan as NT and VU, respectively (Ministry of the Environment, Government of Japan 2019). Furthermore, although *H. japonica* is invasive on some Continents (Van Bocxlaer and Strong 2016), it is also listed on the Japanese Red List as NT (Ministry of the Environment, Government of Japan 2019).

**Figure 1.**
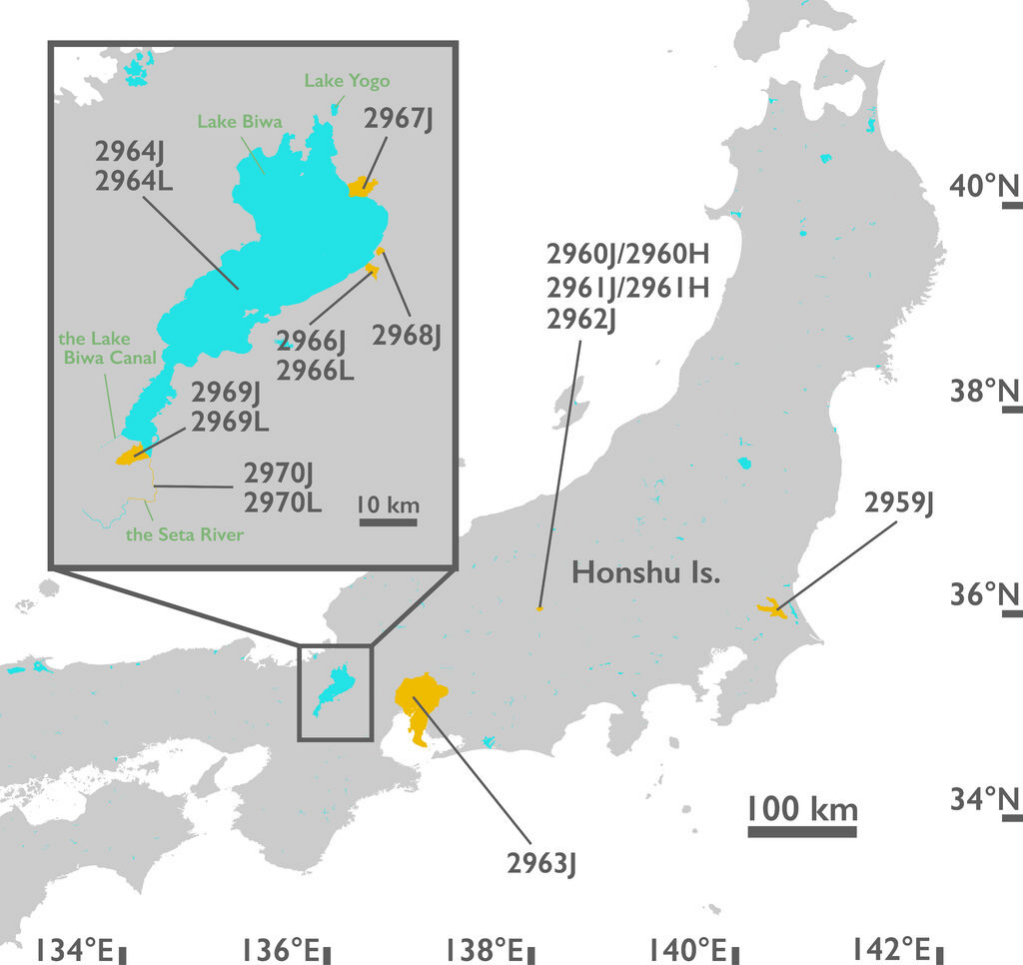
A map of collected localities of *Viviparus scalateri* in Iwakawa's collection . Yellow parts show the estimated ranges, based on the label of the museum lots. Numbers indicate the sample numbers in this study (see also Table 1). Sample numbers 2964J and 2964L were collected from somewhere in Lake Biwa, but the entire Lake is not coloured to make it easier to see. Green letters and downstream of the Seta River are Lake Biwa drainage as defined in this paper. A map is created from digital national land information (Ministry of Land, Infrastructure and Transport of Japan: https://nlftp.mlit.go.jp/ksj/index.html).

The highly-endangered *H. longispira* (Köhler and Rintelen 2011, Nakai 2016, Ministry of the Environment, Government of Japan 2019) is endemic to Lake Biwa drainage (Hirano et al. 2015, Hirano et al. 2019b) which consists of Lake Biwa, a river flowing from Lake Biwa, Lake Yogo (artificially drained from Lake Biwa) and Lake Biwa Canal (Fig. 1). Besides, Lake Biwa has an ancient origin with a remarkable biodiversity (Horie 1971, Yokoyama 1984, Kawanabe 1996, Rossiter 2000, Okuda et al. 2013). Prior to its description, *H. longispira* was included under *Paludina ingallsiana* Lea, 1856 (Kobelt 1879, Iwakawa 1895, Iwakawa 1897a, Iwakawa 1897b). Then, Pilsbry (1902) indicated that *P. ingallsiana* does not occur in Japan and *H. longispira* was included in *Vivipara sclateri* Frauenfeld, 1865. After this, *Vivipara sclateri* (or *Viviparus sclateri*) was adopted as the species name for specimens from Lake Biwa (Hirase 1909, Hirase 1910, Kobelt 1909, Lake Biwa fisheries experimental station 1915, Annandale 1916, Kawamura 1918, Iwakawa 1919). In addition, the name *Vivipara sclateri* was used for viviparid specimens from Japanese localities other than Lake Biwa (Kobelt 1909, Annandale 1916, Iwakawa 1919). Furthermore, Annandale (1921) pointed out the difference between *Vivipara sclateri* by Frauenfeld and the distinctive viviparid gastropods from Lake Biwa (= *H. longispira*), based on comparison with the type illustration of *Vivipara sclateri* and he described specimens from Lake Biwa as *Heterogen turris* Annandale, 1921. However, Smith (1886) had already described the endemic species in Lake Biwa as *Viviparus longispira* Smith, 1886, although this paper was not referred to by other malacologists at that time. Finally, Kuroda (1929) reclassified *Viviparus longispira* as *H. turris* and considered *Viviparus sclateri* to represent a regional subspecies of *H. japonica*, based on the type illustration of *V. sclateri*. Later, *Vivipara sclateri* was generally considered a junior synonym of *H. japonica* (Yagura 1935, Kuroda 1947b, Kuroda 1955, Kuroda 1963). As Annandale (1921) indicated, *H. longispira* is clearly different from typical *Vivipara sclateri*; however, the early malacologists have considered the species to occur in other regions beyond Lake Biwa (Kobelt 1879, Iwakawa 1895, Iwakawa 1897a, Iwakawa 1897b, Iwakawa 1919, Pilsbry 1902, Annandale 1916), although this conclusion remains uncertain. Iwakawa (1919) listed 12 museum lots from 10 localities labelled with 'Naga-tanishi', the current Japanese name for *H. longispira*, which was at the time equivalent to *Viviparus sclateri* and three of the 10 localities are outside Lake Biwa drainage. Although Kuroda (1929) said "these may be elongated *H. japonica*" without any examination, these “*H. longispira*” from areas outside Lake Biwa drainage in Iwakawa (1919) have not been sufficiently examined and have not been illustrated to date. In addition, there were two records of "*H. longispira*" from the southern part of Lake Biwa and the Seta River flowing from Lake Biwa; however, *H. longispira* is now very rare in this area (Nishino 1991, Kihira et al. 2003, Kihira et al. 2009, Nakai 2016). Furthermore, one locality of “*H. longispira*” was drained and is now terrestrial (Ozawa 2012) and the viviparid gastropods from these localities have not been examined. In this study, to clarify the actual status of “*H. longispira*” (=*Viviparus sclateri*) from areas outside the current distribution and to classify extinct populations, we examined the collection of Tomotaro Iwakawa from the National Museum of Nature and Science, Tokyo.

## Materials and methods

All samples were from the mollusc collection of the National Museum of Nature and Science, Tokyo (NSMT-Mo). Specimens and labels were photographed using a digital single-lens reflex camera with a macro lens. Specimens were compared and identified by T. Saito based on mainly the references included in the full list of each species/subspecies synonymy (Suppl. material 1). In particular, the original descriptions of Japanese viviparid species and following references provided principal criteria for comparison and identification: Annandale (1921), Okada and Kurasawa 1950, Habe 1973, Masuda and Uchiyama (2004), Kihira et al. (2009), Hirano et al. (2015), Hirano et al. (2019a) and Hirano et al. (2019b). In addition, some specimens seemed to have an intermediate morphology and were tentatively identified to the species with which they are morphologically most similar.

The synonymy sections in the following text list only the first references for each combination of generic and specific names. As there are many references to Japanese viviparid species, it was impossible to list them all and also to establish objective quantitative criteria for selection. However, all references having the specific names, *sclateri* and *ingallsiana* from Japan were listed in the synonymy because of the main focus of this paper. To enhance reproducibility, we provide all the references that we examined in the synonymy list of Suppl. material 1.

Shell width (SW) of each specimen was measured using a Vernier micrometer (instrumental error: ± 0.03 mm). Furthermore, to compare quantitatively the shell shape of *Heterogen* species in Iwakawa's collection, we conducted elliptic Fourier (EF) analysis (Kuhl and Giardina 1982). The analysis was performed by Momocs 1.2.9 (Bonhomme et al. 2014) under R. 3.5.1 (R Core Team 2018). The EF coefficients were obtained from the images of 83 adult specimens in Iwakawa’s collection and ten specimens figured in the published references (Frauenfeld 1865, Pilsbry 1902, Kobelt 1909, Annandale 1921). In addition, 143 images of *Heterogen* spp. studied by Hirano et al. (2019b) were also provided by these authors for analysis. The images were binarised before tracing shell outlines and the subsequent geometric normalisation was performed with Momocs. The number of harmonics was set to 40. Then, to summarise the results, a principal component analysis (PCA) was conducted using the obtained EF coefficients. Finally, the results of PCA and SW were graphed using Momocs and ggplot2 (Wickham 2016).

## Data resources

Eleven out of 12 lots listed in Iwakawa (1919) were preserved in the National Museum and each lot contained several specimens (Fig. 1 and Table 1). No. 2965 from Kaizu, Omi [Kaizu, Takashima City, Shiga Pref.] in Iwakawa’s catalogue (Iwakawa 1919) was not found in this investigation. To avoid confusion, we separated lots that contained several species/subspecies to species/subspecies-specific lots with modified sample numbers (Table 1). In general, a record No. in the Iwakawa catalogue (Iwakawa 1919) and the museum's registration No. (NSMT-Mo) match. There were two different records having the same catalogue No. 2950 in Iwakawa’s catalogue (Iwakawa 1919): one record was *Viviparus japonicus iwakawa* Pilsbry, 1902 from Nagahama, Iwashiro [Nagahama, Inawashiro City, Fukushima Pref.] and the other was *Viviparus sclateri* from Kasumigaura, Hitachi [Lake Kasumigaura, Ibaraki Pref.]. In addition, no record of No. 2959 existed in the catalogue. In the NSMT collection, there are NSMT-Mo 2950 without location data and NSMT-Mo 2959 from Kasumigaura instead of two 2950 records. Despite the lack of location data, the NSMT-Mo 2950 lot includes morphological *iwakawa* specimens, which are considered *Viviparus japonicus iwakawa* having No. 2950 from Nagahama, Iwashiro. Moreover, the NMST-Mo 2959 lot was labelled with *Viviparus scalateri* and its collected locality was Kasumigaura. Accordingly, we presumed NSMT-Mo 2959 from Kasumigaura as one 2950 record, *Viviparus sclateri* from Kasumigaura, in Iwakawa’s catalogue (Iwakawa 1919). Finally, all specimens information was deposited in the GBIF as an occurrence dataset (https://doi.org/10.15468/mm7yye).

## Taxon treatments

### Heterogen japonica

(Martens, 1861)

43E89D54-0C80-57F6-9581-54279C0754E9

Paludina japonica von Martens 1861 : 44. Type locality: Japan. (original description).
Vivipara sclateri Frauenfeld 1865: 531, pl. 22, unnumbered figs. Type locality: Japan. (original description); Pilsbry 1895: 158; Kobelt 1909: 102, pl. 16, figs. 3–6, 8 [part; pl. 16, fig. 9 and pl. 17, figs. 3 and 5 seemingly have the intermediate morphology between *H. japonica* and *H. longispira*, see discussion]; Annandale 1921: 46 [part].
Paludina ingallsiana — Kobelt 1879: 124, pl. 10, fig. 14 [part; pl. 10, figs. 15–16 and 18 seemingly have intermediate morphology between *H. japonica* and *H. longispira*, see discussion]; Iwakawa 1895: 412, pl. 19, figs. 8–9 [part]; Iwakawa 1897a: 86, pl. 5, fig. 7 [part] (Japanese viviparid catalogue); Iwakawa 1897b: 5.
Paludina oxytropis — Kobelt 1879: 123, pl. 11, fig. 6.
Paludina sclateri — Kobelt 1879: 121, pl. 11, fig. 3; Tanba et al. 1883: 368; Tanba et al. 1891: 216; Iwakawa 1895: 357, pl. 19, fig. 2; Kitahara 1895: 89.
Paludina oxytropis var. japonica — Iwakawa 1897a: 88, pl. 5, figs. 15 and 17 [Iwakawa noted that fig. 15 was an intermediate form between *P. oxytropis oxytropis* and *P. o. japonica*]; Iwakawa 1897b: 9, pl. 2, fig. 13.
Paludina oxytropis var. sclateri — Iwakawa 1897a: 89, pl. 5, figs. 13–14 and 16 [Iwakawa noted that figs. 13 and 16 were an intermediate form between *P. oxytropis oxytropis* and *P. o. sclateri*]; Iwakawa 1897b: 9, pl. 2, figs. 7–9 and 11.
Vivipara oxytropis — Pilsbry 1895: 158.
Viviparus japonicus — Pilsbry 1902: 117, pl. 9, fig. 1.
Viviparus japonicus var. iwakawa Pilsbry 1902: pl. 9, fig. 3. Type locality: Furukawa, Rikuzen [Furukawa City, Miyagi Pref., Japan]. (original description).
Viviparus sclateri — Pilsbry 1902: 118 [part]; Iwakawa 1919: 72 [part].
Vivipara japonica — Kobelt 1909: 99, pl. 15, figs. 1–4.
Vivipara japonica var. iwakawa — Kobelt 1909: 100, pl. 15, figs. 5–7.
Idiopoma (Idiopoma) japonica — Hannibal 1912: 194.
Viviparus japonicus iwakawa — Iwakawa 1919: 71–72.
Viviparus japonecus — *Kanamaru 1920*: 4, pl. 2, fig. 85. [sic].
Lecythoconcha japonica — Annandale 1921: 401.
Lecythoconcha sclateri — Annandale 1921: 401, fig.3; Annandale 1922: 133.
Viviparus (Idiopoma) japonicus — Hirase 1927: 1380, fig. 2653.
Viviparus (Idiopoma) japonicus iwakawa — Hirase 1927: 1381, fig. 2654.
Vivipara japonica var. iwakawae — Prashad 1928: 173, pl. 19, fig. 12. [sic].
Viviparus (Cipangopaludina) japonicus — Prashad 1928: 172.
Viviparus (Dactylochlamys) iwakawae — *Prashad 1928*: 173, pl. 19, fig. 12. [sic].
Viviparus (Viviparus) sclateri — Prashad 1928: 172, pl. 19, fig. 5 [part?].
Viviparus (Cipangopaludina) japonicus iwakawa — Kuroda 1929: 102.
Viviparus (Cipangopaludina) japonicus sclateri — Kuroda 1929: 102; Yagura 1932: 28.
Viviparus oxytropis — Taki 1933 in Horikoshi and Itabashi 1994: 20.
Viviparus iwakawai — *Taki 1946*: 202, fig. 8 [sic].
Cipangopaludina japonica — Hirase and Kuroda 1947c: 1161, fig. 3304; Kuroda 1947b: 3.
Cipangopaludina japonica iwakawa — Hirase and Kuroda 1947a: 1162, fig. 3305.
Cipangopaludina (*Ussuriensis*?) *japonica* — Habe 1990: 4.
Heterogen japonica — Hirano et al. 2019b: 5033, figs. 1, 4–5 and 8.

#### Materials

- **Type status:** Other material. **Occurrence:** occurrenceDetails: https://www.gbif.org/occurrence/2864965303; catalogNumber: 2959J ex.NSMT-Mo 2959; occurrenceRemarks: NMST-Mo 2959 from Kasumigaura was presumed as one record of 2950, *Viviparus sclateri* from Kasumigaura in Iwakawa’s catalogue (1919) (see data resources).; recordNumber: Probably 2950 in Iwakawa 1919; individualCount: 3; lifeStage: adults; preparations: shell (dried); previousIdentifications: *Viviparus sclateri* (Frauenfeld, 1865) in Tomotaro Iwakakawa. 1919. Catalogue of Japanese Mollusca in the Natural history department, Tokyo Imperial Museum, the Tokyo Imperial Museum, Tokyo, Japan, p. 72.; occurrenceID: paper:SK2020:2959J; **Taxon:** scientificName: *Heterogen japonica* (Martens, 1860); kingdom: Animalia; phylum: Mollusca; family: Viviparidae; vernacularName: Oh-tanishi (in Japanese name); **Location:** country: Japan; stateProvince: Ibaraki; locality: Lake Kasumigaura; verbatimLocality: Kasumigaura (in Japanese) [Lake Kasumigaura]; **Identification:** identifiedBy: Takumi Saito; dateIdentified: 2020-02-03/04; **Event:** year: before 1919; **Record Level:** language: Japanese; bibliographicCitation: Tomotaro Iwakakawa. 1919. Catalogue of Japanese Mollusca in the Natural history department, Tokyo Imperial Museum, the Tokyo Imperial Museum, Tokyo, Japan, p. 72.; institutionCode: NSMT; collectionCode: Mo; basisOfRecord: PreservedSpecimen
- **Type status:** Other material. **Occurrence:** occurrenceDetails: https://www.gbif.org/occurrence/2864965315; catalogNumber: 2960J ex.NSMT-Mo 2960; recordNumber: 2960 in Iwakawa 1919; individualCount: 1; lifeStage: adult; preparations: shell (dried); previousIdentifications: *Viviparus sclateri* (Frauenfeld, 1865) in Tomotaro Iwakakawa. 1919. Catalogue of Japanese Mollusca in the Natural history department, Tokyo Imperial Museum, the Tokyo Imperial Museum, Tokyo, Japan, p. 72.; occurrenceID: paper:SK2020:2960J; **Taxon:** scientificName: *Heterogen japonica* (Martens, 1860); kingdom: Animalia; phylum: Mollusca; family: Viviparidae; vernacularName: Oh-tanishi (in Japanese name); **Location:** country: Japan; stateProvince: Nagano; locality: Lake Suwa; verbatimLocality: Suwako (in Japanese) [Lake Suwa]; **Identification:** identifiedBy: Takumi Saito; dateIdentified: 2020-02-03/04; **Event:** year: before 1919; **Record Level:** language: Japanese; bibliographicCitation: Tomotaro Iwakakawa. 1919. Catalogue of Japanese Mollusca in the Natural history department, Tokyo Imperial Museum, the Tokyo Imperial Museum, Tokyo, Japan, p. 72.; institutionCode: NSMT; collectionCode: Mo; basisOfRecord: PreservedSpecimen
- **Type status:** Other material. **Occurrence:** occurrenceDetails: https://www.gbif.org/occurrence/2864965316; catalogNumber: 2961J ex.NSMT-Mo 2961; recordNumber: 2961 in Iwakawa 1919; individualCount: 27; preparations: shell (dried); previousIdentifications: *Viviparus sclateri* (Frauenfeld, 1865) in Tomotaro Iwakakawa. 1919. Catalogue of Japanese Mollusca in the Natural history department, Tokyo Imperial Museum, the Tokyo Imperial Museum, Tokyo, Japan, p. 72.; occurrenceID: paper:SK2020:2961J; **Taxon:** scientificName: *Heterogen japonica* (Martens, 1860); kingdom: Animalia; phylum: Mollusca; family: Viviparidae; vernacularName: Oh-tanishi (in Japanese name); **Location:** country: Japan; stateProvince: Nagano; locality: Lake Suwa; verbatimLocality: Suwako (in Japanese) [Lake Suwa]; **Identification:** identifiedBy: Takumi Saito; dateIdentified: 2020-02-03/04; **Event:** year: before 1919; **Record Level:** language: Japanese; bibliographicCitation: Tomotaro Iwakakawa. 1919. Catalogue of Japanese Mollusca in the Natural history department, Tokyo Imperial Museum, the Tokyo Imperial Museum, Tokyo, Japan, p. 72.; institutionCode: NSMT; collectionCode: Mo; basisOfRecord: PreservedSpecimen
- **Type status:** Other material. **Occurrence:** occurrenceDetails: https://www.gbif.org/occurrence/2864965304; catalogNumber: 2962J ex.NSMT-Mo 2962; recordNumber: 2962 in Iwakawa 1919; individualCount: 6; lifeStage: adult; preparations: shell (dried); previousIdentifications: *Viviparus sclateri* (Frauenfeld, 1865) in Tomotaro Iwakakawa. 1919. Catalogue of Japanese Mollusca in the Natural history department, Tokyo Imperial Museum, the Tokyo Imperial Museum, Tokyo, Japan, p. 72.; occurrenceID: paper:SK2020:2962J; **Taxon:** scientificName: *Heterogen japonica* (Martens, 1860); kingdom: Animalia; phylum: Mollusca; family: Viviparidae; vernacularName: Oh-tanishi (in Japanese name); **Location:** country: Japan; stateProvince: Nagano; locality: Lake Suwa; verbatimLocality: Tenryu-gawa-no-suigen (in Japanese) [The water source of Tenryu River = Lake Suwa]; **Identification:** identifiedBy: Takumi Saito; dateIdentified: 2020-02-03/04; **Event:** year: before 1919; **Record Level:** language: Japanese; bibliographicCitation: Tomotaro Iwakakawa. 1919. Catalogue of Japanese Mollusca in the Natural history department, Tokyo Imperial Museum, the Tokyo Imperial Museum, Tokyo, Japan, p. 72.; institutionCode: NSMT; collectionCode: Mo; basisOfRecord: PreservedSpecimen
- **Type status:** Other material. **Occurrence:** occurrenceDetails: https://www.gbif.org/occurrence/2864965307; catalogNumber: 2963J ex.NSMT-Mo 2963; recordNumber: 2963 in Iwakawa 1919; individualCount: 1; lifeStage: adult; preparations: shell (dried); previousIdentifications: *Viviparus sclateri* (Frauenfeld, 1865) in Tomotaro Iwakakawa. 1919. Catalogue of Japanese Mollusca in the Natural history department, Tokyo Imperial Museum, the Tokyo Imperial Museum, Tokyo, Japan, p. 72.; occurrenceID: paper:SK2020:2963J; **Taxon:** scientificName: *Heterogen japonica* (Martens, 1860); kingdom: Animalia; phylum: Mollusca; family: Viviparidae; vernacularName: Oh-tanishi (in Japanese name); **Location:** country: Japan; stateProvince: Aichi; locality: western part of Aichi Prefectue; verbatimLocality: Owari (in Japanese) [Old name of western part of Aichi Prefectue]; **Identification:** identifiedBy: Takumi Saito; dateIdentified: 2020-02-03/04; **Event:** year: before 1919; **Record Level:** language: Japanese; bibliographicCitation: Tomotaro Iwakakawa. 1919. Catalogue of Japanese Mollusca in the Natural history department, Tokyo Imperial Museum, the Tokyo Imperial Museum, Tokyo, Japan, p. 72.; institutionCode: NSMT; collectionCode: Mo; basisOfRecord: PreservedSpecimen
- **Type status:** Other material. **Occurrence:** occurrenceDetails: https://www.gbif.org/occurrence/2864965302; catalogNumber: 2964J ex.NSMT-Mo 2964; recordNumber: 2964 in Iwakawa 1919; individualCount: 1; lifeStage: adult; preparations: shell (dried); previousIdentifications: *Viviparus sclateri* (Frauenfeld, 1865) in Tomotaro Iwakakawa. 1919. Catalogue of Japanese Mollusca in the Natural history department, Tokyo Imperial Museum, the Tokyo Imperial Museum, Tokyo, Japan, p. 72.; occurrenceID: paper:SK2020:2964J; **Taxon:** scientificName: *Heterogen japonica* (Martens, 1860); kingdom: Animalia; phylum: Mollusca; family: Viviparidae; vernacularName: Oh-tanishi (in Japanese name); **Location:** country: Japan; stateProvince: Shiga; locality: Lakw Biwa; verbatimLocality: Biwa-ko (in Japanese) [Lake Biwa]; **Identification:** identifiedBy: Takumi Saito; dateIdentified: 2020-02-03/04; **Event:** year: before 1919; **Record Level:** language: Japanese; bibliographicCitation: Tomotaro Iwakakawa. 1919. Catalogue of Japanese Mollusca in the Natural history department, Tokyo Imperial Museum, the Tokyo Imperial Museum, Tokyo, Japan, p. 72.; institutionCode: NSMT; collectionCode: Mo; basisOfRecord: PreservedSpecimen
- **Type status:** Other material. **Occurrence:** occurrenceDetails: https://www.gbif.org/occurrence/2864965311; catalogNumber: 2966J ex.NSMT-Mo 2966; recordNumber: 2966 in Iwakawa 1919; individualCount: 1; lifeStage: adult; preparations: shell (dried); previousIdentifications: *Viviparus sclateri* (Frauenfeld, 1865) in Tomotaro Iwakakawa. 1919. Catalogue of Japanese Mollusca in the Natural history department, Tokyo Imperial Museum, the Tokyo Imperial Museum, Tokyo, Japan, p. 72.; occurrenceID: paper:SK2020:2966J; **Taxon:** scientificName: *Heterogen japonica* (Martens, 1860); kingdom: Animalia; phylum: Mollusca; family: Viviparidae; vernacularName: Oh-tanishi (in Japanese name); **Location:** country: Japan; stateProvince: Shiga; locality: Hikone, Hikone City; verbatimLocality: Hikone (in Japanese) [Hikone, Hikone City]; **Identification:** identifiedBy: Takumi Saito; dateIdentified: 2020-02-03/04; **Event:** year: before 1919; **Record Level:** language: Japanese; bibliographicCitation: Tomotaro Iwakakawa. 1919. Catalogue of Japanese Mollusca in the Natural history department, Tokyo Imperial Museum, the Tokyo Imperial Museum, Tokyo, Japan, p. 72.; institutionCode: NSMT; collectionCode: Mo; basisOfRecord: PreservedSpecimen
- **Type status:** Other material. **Occurrence:** occurrenceDetails: https://www.gbif.org/occurrence/2864965312; catalogNumber: 2967J ex.NSMT-Mo 2967; recordNumber: 2967 in Iwakawa 1919; individualCount: 7; lifeStage: adults; preparations: shell (dried); previousIdentifications: *Viviparus sclateri* (Frauenfeld, 1865) in Tomotaro Iwakakawa. 1919. Catalogue of Japanese Mollusca in the Natural history department, Tokyo Imperial Museum, the Tokyo Imperial Museum, Tokyo, Japan, p. 72.; occurrenceID: paper:SK2020:2967J; **Taxon:** scientificName: *Heterogen japonica* (Martens, 1860); kingdom: Animalia; phylum: Mollusca; family: Viviparidae; vernacularName: Oh-tanishi (in Japanese name); **Location:** country: Japan; stateProvince: Shiga; locality: Maibara City, Irie; verbatimLocality: Irie-mura (in Japanese) [Irie Village, old name of Irie, Maibara City]; **Identification:** identifiedBy: Takumi Saito; dateIdentified: 2020-02-03/04; **Event:** year: before 1919; **Record Level:** language: Japanese; bibliographicCitation: Tomotaro Iwakakawa. 1919. Catalogue of Japanese Mollusca in the Natural history department, Tokyo Imperial Museum, the Tokyo Imperial Museum, Tokyo, Japan, p. 72.; institutionCode: NSMT; collectionCode: Mo; basisOfRecord: PreservedSpecimen
- **Type status:** Other material. **Occurrence:** occurrenceDetails: https://www.gbif.org/occurrence/2864965309; catalogNumber: 2968J ex.NSMT-Mo 2968; recordNumber: 2968 in Iwakawa 1919; individualCount: 15; lifeStage: 13 adults 2 young; preparations: shell (dried); previousIdentifications: *Viviparus sclateri* (Frauenfeld, 1865) in Tomotaro Iwakakawa. 1919. Catalogue of Japanese Mollusca in the Natural history department, Tokyo Imperial Museum, the Tokyo Imperial Museum, Tokyo, Japan, p. 72.; occurrenceID: paper:SK2020:2968J; **Taxon:** scientificName: *Heterogen japonica* (Martens, 1860); kingdom: Animalia; phylum: Mollusca; family: Viviparidae; vernacularName: Oh-tanishi (in Japanese name); **Location:** country: Japan; stateProvince: Shiga; locality: Tsukuma Ragoon; verbatimLocality: Tsukuma-uchiko (in Japanese) [Tsukuma Ragoon]; **Identification:** identifiedBy: Takumi Saito; dateIdentified: 2020-02-03/04; **Event:** year: before 1919; **Record Level:** language: Japanese; bibliographicCitation: Tomotaro Iwakakawa. 1919. Catalogue of Japanese Mollusca in the Natural history department, Tokyo Imperial Museum, the Tokyo Imperial Museum, Tokyo, Japan, p. 72.; institutionCode: NSMT; collectionCode: Mo; basisOfRecord: PreservedSpecimen
- **Type status:** Other material. **Occurrence:** occurrenceDetails: https://www.gbif.org/occurrence/2864965310; catalogNumber: 2969J ex.NSMT-Mo 2969; recordNumber: 2969 in Iwakawa 1919; individualCount: 4; lifeStage: 3 adults 1 young; preparations: shell (dried); previousIdentifications: *Viviparus sclateri* (Frauenfeld, 1865) in Tomotaro Iwakakawa. 1919. Catalogue of Japanese Mollusca in the Natural history department, Tokyo Imperial Museum, the Tokyo Imperial Museum, Tokyo, Japan, p. 72.; occurrenceID: paper:SK2020:2969J; **Taxon:** scientificName: *Heterogen japonica* (Martens, 1860); kingdom: Animalia; phylum: Mollusca; family: Viviparidae; vernacularName: Oh-tanishi (in Japanese name); **Location:** country: Japan; stateProvince: Shiga; locality: Ohtsu City, Zeze; verbatimLocality: Zeze (in Japanese) [Zeze, Ohtsu City]; **Identification:** identifiedBy: Takumi Saito; dateIdentified: 2020-02-03/04; identificationRemarks: Some specimens seemingly have the intermediate morphology between *H. japonica* and *H. longispira* (see taxon discussion section).; **Event:** year: before 1919; **Record Level:** language: japanese; bibliographicCitation: Tomotaro Iwakakawa. 1919. Catalogue of Japanese Mollusca in the Natural history department, Tokyo Imperial Museum, the Tokyo Imperial Museum, Tokyo, Japan, p. 72.; institutionCode: NSMT; collectionCode: Mo; basisOfRecord: PreservedSpecimen
- **Type status:** Other material. **Occurrence:** occurrenceDetails: https://www.gbif.org/occurrence/2864965317; catalogNumber: 2970J ex.NSMT-Mo 2970; recordNumber: 2970 in Iwakawa 1919; individualCount: 2; lifeStage: 2 juveniles; preparations: shell (dried); previousIdentifications: *Viviparus sclateri* (Frauenfeld, 1865) in Tomotaro Iwakakawa. 1919. Catalogue of Japanese Mollusca in the Natural history department, Tokyo Imperial Museum, the Tokyo Imperial Museum, Tokyo, Japan, p. 72.; occurrenceID: paper:SK2020:2970J; **Taxon:** scientificName: *Heterogen japonica* (Martens, 1860); kingdom: Animalia; phylum: Mollusca; family: Viviparidae; vernacularName: Oh-tanishi (in Japanese name); **Location:** country: Japan; stateProvince: Shiga; locality: theSeta River; verbatimLocality: Seta-gawa (in Japanese) [the Seta River]; **Identification:** identifiedBy: Takumi Saito; dateIdentified: 2020-02-03/04; identificationRemarks: Some specimens seemingly have the intermediate morphology between *H. japonica* and *H. longispira* (see taxon discussion section).; **Event:** year: before 1919; **Record Level:** language: Japanese; bibliographicCitation: Tomotaro Iwakakawa. 1919. Catalogue of Japanese Mollusca in the Natural history department, Tokyo Imperial Museum, the Tokyo Imperial Museum, Tokyo, Japan, p. 72.; institutionCode: NSMT; collectionCode: Mo; basisOfRecord: PreservedSpecimen

#### Diagnosis

Adult shell large and thick; shell shape subconical to pyramidal. Adult shell colour dark brown or brown or dark olive, often covered with many environmental attachments, such as alga; shell surface glossy, sometimes having weak hollows, growth lines and spiral striae. Suture moderately deep. Young shell small, relatively thin and fragile; shell shape pyramidal. Young shell colour yellowish-olive; shell surface quite glossy with no spiral ridges on the upper part of the spire.

#### Taxon discussion

*Heterogen japonica* can be distinguished from other Japanese Viviparidae by the following criteria which are based on the illustrations and information from literature (Suppl. material 1).

**Figure 2.**
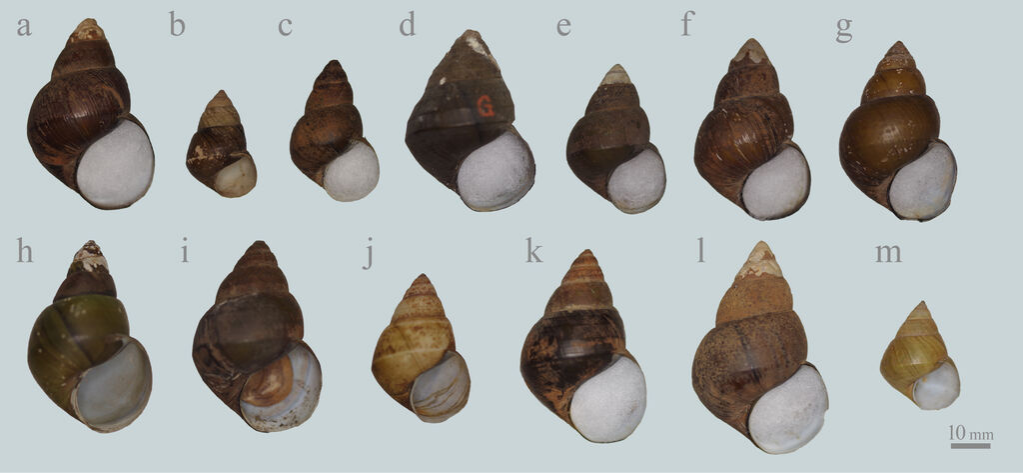
Representative shells of *Heterogen japonica* (Martens, 1861) from Japan, deposited in the collection of the National Museum of Nature and Science, Tokyo, which were treated as *Viviparus sclateri* (Frauenfeld, 1865) in Iwakawa (1919). **a**: 2959J from Lake Kasumigaura, Ibaraki Pref. **b-g**: specimens from Lake Suwa, Nagano Pref. (b: 2960J, c-e: 2961J, f-g: 2962J). **h**: 2963J from the western part of Aichi Pref. **i**: 2964J from Lake Biwa, Shiga Pref. **j**: 2966J from Hikone City, Shiga Pref. **k**: 2967J from Maibara City, Shiga Pref. **l-m**: 2968J from Tsukuma Lagoon, Shiga Pref. (l: adult, m: young).

*H. japonica* can basically be distinguished from *Cipangopaludina chinensis laeta* by its subconical to pyramidal shell shape (Kihira et al. 2009; Fig. 2). In addition, the spire is higher, the spire angle is narrower and the shell apex is more pointed than those of *C. c. laeta.* Furthermore, *C. c. laeta* often has a lipped aperture, but the aperture of *H. japonica* does not have a pronounced lip (Masuda and Uchiyama 2004). Next, the differences with *C. chinensis chinensis* are not fully revealed, as the shell morphology of *C. c. chinensis* in Japan was not examined in depth. Nevertheless, based on the analysis and illustration of Hirano et al. (2019a), the only study that explicitly examined Japanese *C. c. chinensis*, the shell shape of *C. c. chinensis* is similar to that of *C. c. laeta* except for the pointed shell apex of *C. c. chinensis.* In any case, *C. c. chinensis* is distributed only around Kyushu Island, the south-western part of Japan (Hirano et al. 2019a) and no specimens of this subspecies were included in our study.

Adult *Sinotaia quadrata histrica* is generally smaller than *H. japonica*. There is a high morphological diversity in the shell shape of *S. q. histrica* (Kihira et al. 2009); however, the body whorl and the spire whorl are more rounded and arched than that of *H. japonica*. In addition, the shell apex of *S. q. histrica* is more rounded and the aperture is relatively smaller than those of specimens of *H. japonica* having the similar shell shape, as the spire is dense. Moreover, the shell colour of *S. q. histrica* seems to be brighter than that in *H. japonica* and often has a yellowish colour.

*H. japonica* differs in shell shape from *H. longispira* (Hirano et al. 2019b). Quantitatively, *H. japonica* has a lower spire and a broader spiral angle than *H. longispira* (Kihira et al. 2009). In addition, *H. longispira* has a strong basal angulation even on the adult shell (Okada and Kurasawa 1950). Furthermore, the body whorl and the spire whorl of *H. longispira* are linear (like vertical) in lateral view and the upper periphery of the whorls turns sharply to nearly horizontal. Accordingly, the suture is quite deep and whorls have a strong shoulder just below the suture. This feature is extremely pronounced in juveniles and young shells and this morphological difference is diagnostic between two species (Okada and Kurasawa 1950, Hirano et al. 2019b). In addition, a difference that rarely appears in the shell shape is the distinct spiral ridges on the upper whorl of adult *H. longspira* (Hirano et al. 2019b). The early whorl of adult *H. japonica* does not have such spiral ridges. Moreover, *Heterogen* sp. has weak spiral ridges on the upper whorls and so this is a distinguishing feature from *Heterogen* sp., which is not distinguished by the shell shape (Hirano et al. 2019b). The spiral ridges of *H. longispira* are also pronounced on the body whorl of the adult shell. Furthermore, some *H. japonica* usually has greenish shells without reddish colour (e.g. Fig. 2h), whereas the shell colour of most *H. longispira* contains reddish colour and accordingly are often brown or dark brown in colour.

Besides, *H. japonica* has one morphotype, ver. *iwakawa*, which had been described and synonymised. This morphotype has a pyramidal shell shape, a broad spire angle and a strong basal angulation (e.g. Fig. 2d). Typical specimens of Viviparus japonicus var. iwakawa (Pilsbry 1902) is very easy to distinguish from any other Japanese Viviparidae; however, the morphology is continuous with *H. japonica* (Okada and Kurasawa 1950).

#### Notes

**Figure 3.**
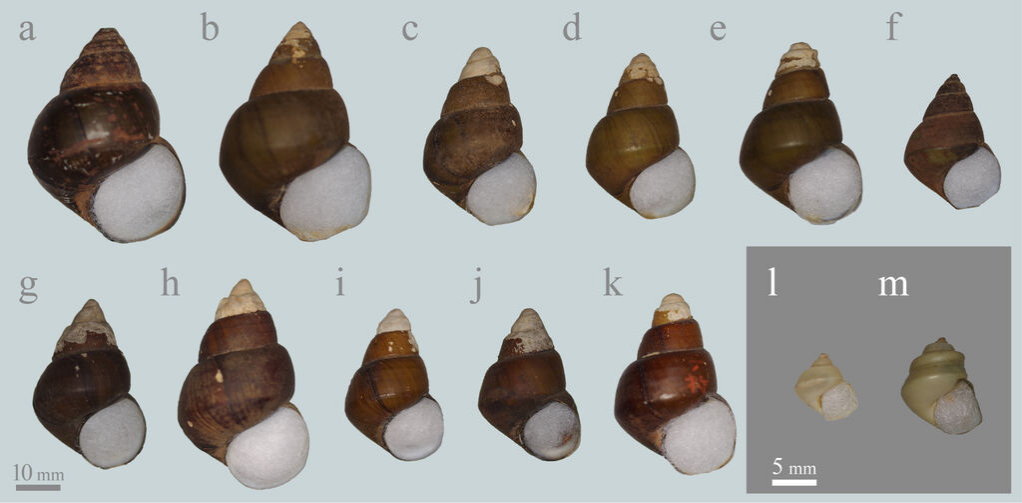
Shells of *Heterogen japonica* (Martens, 1861), *H. longispira* (Smith, 1886), and the intermediate morphology between the two species stored at the National Museum of Nature and Science, Tokyo, which were treated as *Viviparus sclateri* (Frauenfeld, 1865) in Iwakawa's catalogue (1919). **a-b**: *H. japonica* in 2969J from Otsu City, Shiga Pref. **c-d**: non-typical *H. longispira* in 2969L from Otsu City, Shiga Pref. **e**: typical *H. longispira* in 2969L from Otsu City, Shiga Pref. **f**: subadult *H. japonica* in 2969J from Otsu City, Shiga Pref. **g-j**: non-typical and intermediate *H. longispira* in 2970L from the Seta River, Shiga Pref. **K**: typical *H. longispira* in 2970L from the Seta River, Shiga Pref. **l**: a juvenile shell of *H. japonica* in 2970J from the Seta River, Shiga Pref. **m**: a typical juvenile shell of *H. longispira* in 2970L from the Seta River, Shiga Pref.

*Heterogen japonica* was identified in all 11 studied lots (Figs 2, 3). Some specimens (Fig. 2b-c, h and l) have the high spire and the narrow spire angle and the shell shapes of these specimens are relatively similar to *H. longispira*. However, specimens assigned to *H. japonica* lack a lot of the distinctive features *of H. longispira*. Lots Mo2969 and Mo2970 contained specimens with an intermediate morphology between *H. japonica* and *H. longispira* (e.g. Fig. 3c and g-h). Therefore, in this study, we treated only specimens that were clearly distinguished from *H. longispira* (e.g. Fig. 3a-b and l) as *H. japonica*.

Based on the previous taxonomy (Habe 1973), synonymy included species-group name, *iwakawa* (and its mandatory changes in spelling and incorrectsubsequentspellings) in addition to species-group name, *japonica* (and its mandatory changes in spelling and incorrectsubsequentspellings). Furthermore, since *Paludina oxytropis* in Kobelt (1879) and *Vivipara oxytropis* in Pilsbry (1895) were later synonymised by the authors themselves (Kobelt 1909, Pilsbry 1902), these were also listed in synonymy. As to the species-group names *ingallsiana* and *sclateri*, we refer to the discussion.

### Heterogen longispira

(Smith, 1886)

3B035A91-356C-56D2-93AB-05E0A55E53CD

Paludina ingallsiana — Kobelt 1879: 124, pl. 10, figs. 15–18, pl. 11, fig. 2 [part; pl. 10, figs. 15–16 and 18 seemingly have the intermediate morphology between *H. japonica* and *H. longispira*, see discussion]; Tanba et al. 1883: 368; Iwakawa 1895: 412 [part]; Iwakawa 1897a: 86 [part]; Iwakawa 1897b: 5 [part].
Paludina longispira Smith 1886: 57–58. Type locality: Lake Biwa. (original description).
Viviparus sclateri — Pilsbry 1902: 118, pl.9, fig. 4 [part; see discussion]; Hirase 1909: 45 [part?]; Hirase 1910: 15 [part?]; Lake Biwa fisheries experimental station 1915: 30, fig. 13; Iwakawa 1919: 72 [part].
Vivipara sclateri — Kobelt 1909: 102, pl. 16, figs. 7, 9, pl. 17, figs. 1–5. [part; pl. 16, fig. 9 and pl. 17, figs. 3, 5 seemingly have the intermediate morphology between *H. japonica* and *H. longispira*, see discussion]; Annandale 1916: 46 [part]; Kawamura 1918: 358, fig. 441 [part?].
Heterogen turris Annandale 1921: 400, figs. 1–2. Type locality: Lake Biwa. (original description).
Viviparus (Heterogen) turris — Hirase 1927: 1381, fig. 2655.
Viviparus (Heterogen) longispira — Prashad 1928: 172, pl. 19, fig. 7.
Heterogen longispira — Kuroda 1947b: 3.
Viviparus (Heterogen) turis — Okada and Kurasawa 1950: 153, figs. 10–11, and 14, (in text), pl. 2, figs. 7–8, pl. 3, figs. 9–10, pl. 4, figs. a"–g". [sic].
Viviparus longispira — Hirase and Taki 1951: pl. 77, fig. 10.
Cipangopaludina (Heterogen) longispira — Kuroda and Habe 1965a: 48, fig. 151.

#### Materials

- **Type status:** Other material. **Occurrence:** occurrenceDetails: https://www.gbif.org/occurrence/2864965305; catalogNumber: 2964L ex.NSMT-Mo 2964; recordNumber: 2964 in Iwakawa 1919; individualCount: 1; lifeStage: adult; preparations: shell (dried); previousIdentifications: *Viviparus sclateri* (Frauenfeld, 1865) in Tomotaro Iwakakawa. 1919. Catalogue of Japanese Mollusca in the Natural history department, Tokyo Imperial Museum, the tokyo Imperial Museum, Tokyo, Japan, p. 72.; occurrenceID: paper:SK2020:2964L; **Taxon:** scientificName: *Heterogen longispira* (Smith, 1886); kingdom: Animalia; phylum: Mollusca; family: Viviparidae; vernacularName: Naga-tanishi (in Japanese name); **Location:** country: Japan; stateProvince: Shiga; locality: Lake Biwa; verbatimLocality: Biwa-ko (in Japanese) [Lake Biwa]; **Identification:** identifiedBy: Takumi Saito; dateIdentified: 2020-02-03/04; **Event:** year: before 1919; **Record Level:** language: Japanese; bibliographicCitation: Tomotaro Iwakakawa. 1919. Catalogue of Japanese Mollusca in the Natural history department, Tokyo Imperial Museum, the tokyo Imperial Museum, Tokyo, Japan, p. 72.; institutionCode: NSMT; collectionCode: Mo; basisOfRecord: PreservedSpecimen
- **Type status:** Other material. **Occurrence:** occurrenceDetails: https://www.gbif.org/occurrence/2864965303; catalogNumber: 2966L ex.NSMT-Mo 2966; recordNumber: 2966 in Iwakawa 1919; individualCount: 2; lifeStage: 1adult 1 young; preparations: shell (dried); previousIdentifications: *Viviparus sclateri* (Frauenfeld, 1865) in Tomotaro Iwakakawa. 1919. Catalogue of Japanese Mollusca in the Natural history department, Tokyo Imperial Museum, the tokyo Imperial Museum, Tokyo, Japan, p. 72.; occurrenceID: paper:SK2020:2966L; **Taxon:** scientificName: *Heterogen longispira* (Smith, 1886); kingdom: Animalia; phylum: Mollusca; family: Viviparidae; vernacularName: Naga-tanishi (in Japanese name); **Location:** country: Japan; stateProvince: Shiga; locality: Hikone, Hikone City; verbatimLocality: Hikone (in Japanese) [Hikone, Hikone City]; **Identification:** identifiedBy: Takumi Saito; dateIdentified: 2020-02-03/04; **Event:** year: before 1919; **Record Level:** language: Japanese; bibliographicCitation: Tomotaro Iwakakawa. 1919. Catalogue of Japanese Mollusca in the Natural history department, Tokyo Imperial Museum, the tokyo Imperial Museum, Tokyo, Japan, p. 72.; institutionCode: NSMT; collectionCode: Mo; basisOfRecord: PreservedSpecimen
- **Type status:** Other material. **Occurrence:** occurrenceDetails: https://www.gbif.org/occurrence/2864965313; catalogNumber: 2969L ex.NSMT-Mo 2969; recordNumber: 2969 in Iwakawa 1919; individualCount: 6; lifeStage: adults; preparations: shell (dried); previousIdentifications: *Viviparus sclateri* (Frauenfeld, 1865) in Tomotaro Iwakakawa. 1919. Catalogue of Japanese Mollusca in the Natural history department, Tokyo Imperial Museum, the tokyo Imperial Museum, Tokyo, Japan, p. 72.; occurrenceID: paper:SK2020:2969L; **Taxon:** scientificName: *Heterogen longispira* (Smith, 1886); kingdom: Animalia; phylum: Mollusca; family: Viviparidae; vernacularName: Naga-tanishi (in Japanese name); **Location:** country: Japan; stateProvince: Shiga; locality: Zeze, Ohtsu City; verbatimLocality: Zeze (in Japanese) [Zeze, Ohtsu City]; **Identification:** identifiedBy: Takumi Saito; dateIdentified: 2020-02-03/04; identificationRemarks: Some specimens are presumed to be hybrid species between *H. longispira* and *H. japonica* (see taxon discussion section).; **Event:** year: before 1919; **Record Level:** language: Japanese; bibliographicCitation: Tomotaro Iwakakawa. 1919. Catalogue of Japanese Mollusca in the Natural history department, Tokyo Imperial Museum, the tokyo Imperial Museum, Tokyo, Japan, p. 72.; institutionCode: NSMT; collectionCode: Mo; basisOfRecord: PreservedSpecimen
- **Type status:** Other material. **Occurrence:** occurrenceDetails: https://www.gbif.org/occurrence/2864965314; catalogNumber: 2970L ex.NSMT-Mo 2970; recordNumber: 2970 in Iwakawa 1919; individualCount: 15; lifeStage: 11 adults 4 juveniles; preparations: shell (dried); previousIdentifications: *Viviparus sclateri* (Frauenfeld, 1865) in Tomotaro Iwakakawa. 1919. Catalogue of Japanese Mollusca in the Natural history department, Tokyo Imperial Museum, the tokyo Imperial Museum, Tokyo, Japan, p. 72.; occurrenceID: paper:SK2020:2970L; **Taxon:** scientificName: *Heterogen longispira* (Smith, 1886); kingdom: Animalia; phylum: Mollusca; family: Viviparidae; vernacularName: Naga-tanishi (in Japanese name); **Location:** country: Japan; stateProvince: Shiga; locality: Seta River; verbatimLocality: Seta-gawa (in Japanese) [the Seta River]; **Identification:** identifiedBy: Takumi Saito; dateIdentified: 2020-02-03/04; identificationRemarks: Some specimens are presumed to be hybrid species between *H. longispira* and *H. japonica* (see taxon discussion section).; **Event:** year: before 1919; **Record Level:** language: Japanese; bibliographicCitation: Tomotaro Iwakakawa. 1919. Catalogue of Japanese Mollusca in the Natural history department, Tokyo Imperial Museum, the tokyo Imperial Museum, Tokyo, Japan, p. 72.; institutionCode: NSMT; collectionCode: Mo; basisOfRecord: PreservedSpecimen

#### Diagnosis

Adult shell moderately large and very thick; shell shape subconical. Adult shell dark brown or greenish-brown sometimes covered with attachments; shell surface often having growth lines and several spiral ridges. Suture deeper than in any other viviparid from Japan; whorls strongly shouldered. Young shell small and relatively thin, but not fragile; shell shape pyramidal to subconical. Young shell bright olive; shell surface quite glossy having strong spiral ridges on upper part of spire. The shell size upon birth is larger than that of any other viviparid gastropod in Japan.

#### Taxon discussion

*Heterogen longspira* can be distinguished from other Japanese Viviparidae by criteria based on the illustrations and information from literature (Suppl. material 1). In particular, the juvenile and young shell have strong spiral ridges on the upper part of each whorl, this feature having crucial diagnostic value (Okada and Kurasawa 1950, Hirano et al. 2019b). Furthermore, the shell size of the juvenile upon birth is larger than that of any other viviparid gastropods in Japan.

**Figure 4.**
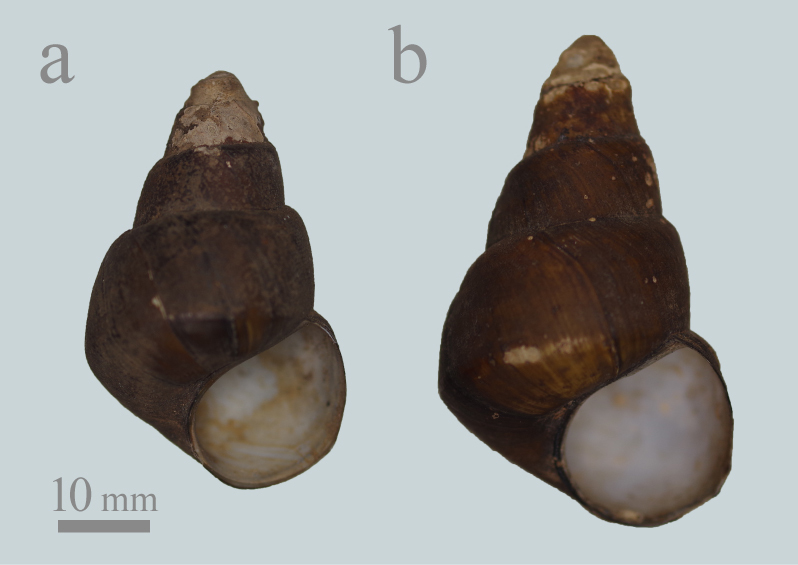
Representative shells of *Heterogen longispira* (Smith, 1886) from Japan, deposited in the collection of the National Museum of Nature and Science, Tokyo, which were treated as *Viviparus sclateri* (Frauenfeld, 1865) in Iwakawa (1919). **a**: 2964L from Lake Biwa, Shiga Pref. **b**: 2966L from Hikone City, Shiga Pref.

Firstly, *H. longispira* is easily distinguished from *Cipangopaludina* species/subspecies in Japan, based on the pyramidal shell shape, the higher spire, the narrower spire angle and the linear body whorl (Kihira et al. 2009; Fig. 4).

Besides, adult *Sinotaia quadrata histrica* is generally smaller than *H. longispira*. There is a high morphological diversity in the shell shape of *S. q. histrica* (Kihira et al. 2009); however, the body whorl and the spire whorl are much more rounded and arched than those of *H. longispira*. In addition, the suture of *S. q. histrica* is shallower than that of *H. longispira*. The upper whorl of adult *S. q. histrica* does not have the pronounced spiral ridges. Moreover, the shell colour of *S. q. histrica* is brighter than that in *H. longispira* and often has a yellowish colour.

Both adult *H. longispira and Heterogen* sp. have the spiral ridges on the upper whorl (Hirano et al. 2019b), but the ridges of *H. longispira* are stronger than *Heterogen* sp. In addition, the shell shape of *Heterogen* sp. is indistinguishable from that of *H. japonica* and then there are some differences in the shell shape between *H. longispira* and *Heterogen* sp. (Hirano et al. 2019b; refer to taxon discussion on the section of *H. japonica* for the difference).

#### Notes

*Heterogen longispira* was identified in four of the 11 lots examined, which were all from Lake Biwa drainage only (Figs 3, 4). Lots Mo2969 and Mo2970 contained non-typical *H. longispira* with an intermediate morphology to *H. japonica* and *H. longispira* (e.g. Fig. 3c and g-k). The shell shapes of these specimens tend to be slightly more similar to *H. japonica*; however, they have several distinctive morphological features of *H. longispira*, namely, the pronounced spiral ridges, the shouldered whorl, the deep suture, the strong basal angle and the high spire . Therefore these specimens were tentatively identified as *H. longispira* here (see also discussion).

Based on the previous taxonomy (Kuroda 1929), synonymy included the species-group name *turris* (and incorrectsubsequentspellings) in addition to the species-group name *longispira*. For species-group names *ingallsiana* and *sclateri*, refer to the discussion.

### Sinotaia quadrata histrica

(Gould, 1859)

0DFC5245-C596-56BE-98BA-6ABCAA58F5F2

Paludina histrica Gould 1859: 41. Type locality: Ousima and Loo Choo [Amami-Oshima and the Ryukyu Islands]. (original description).
[?]*Paludina nitens*Reeve 1863: pl. 10, fig. 59. Type locality: Japan. (original description).
Paludina ingallsiana — Iwakawa 1897a: 86, pl. 5, figs. 5–6 [part]; Iwakawa 1897b: 5, pl. 2, fig. 6 [part].
Viviparus histricus — Pilsbry 1902: pl. 9, fig. 5.
Vivipara histrica — Kobelt 1909: 107, pl. 16, fig. 6.
[?]*Vivipara nitens* — Kobelt 1909: 107.
[?]*Vivipara lacustris* — Kawamura 1918: 358.
Viviparus quadratus var. ? histricus — Hirase 1927: 1382, fig. 2656.
Cipangopaludina histrica — Kuroda 1928: 32.
Viviparus (Viviparus) histricus — Prashad 1928: 172.
Viviparus histricus — Iwakawa 1919: 72.
Viviparus (Sinotaia) histricus — Hirase and Kuroda 1947b: 1160, fig. 3301.
Taia (Sinotaia) histrica — Kuroda 1947b: 4.
Sinotaia histrica — Kuroda 1948: 26.
Viviparus (Idiopoma) histricus — Okada and Kurasawa 1950: 149, figs. 3–4, 12–14 (in text), pl. 1, figs. 1–2, pl. 3, figs. a'–g'.
Sinotaia quadratus histrica — Kuroda and Habe 1965b: 48, fig. 152.
Sinotaia quadrata histrica — Habe and Kosuge 1967: 27, pl. 11, fig. 8.

#### Materials

- **Type status:** Other material. **Occurrence:** occurrenceDetails: https://www.gbif.org/occurrence/2864965306; catalogNumber: 2960H ex.NSMT-Mo 2960; recordNumber: 2960 in Iwakawa 1919; individualCount: 1; lifeStage: adult; preparations: shell (dried); previousIdentifications: *Viviparus sclateri* (Frauenfeld, 1865) in Tomotaro Iwakakawa. 1919. Catalogue of Japanese Mollusca in the Natural history department, Tokyo Imperial Museum, the tokyo Imperial Museum, Tokyo, Japan, p. 72.; occurrenceID: paper:SK2020:2960H; **Taxon:** scientificName: *Sinotaia quadrata histrica* (Gould, 1859); kingdom: Animalia; phylum: Mollusca; family: Viviparidae; vernacularName: Hime-tanishi (in Japanese name); **Location:** country: Japan; stateProvince: Nagano; locality: Lake Suwa; verbatimLocality: Suwako (in Japanese) [Lake Suwa]; **Identification:** identifiedBy: Takumi Saito; dateIdentified: 2020-02-03/04; **Event:** year: before 1919; **Record Level:** language: Japanese; bibliographicCitation: Tomotaro Iwakakawa. 1919. Catalogue of Japanese Mollusca in the Natural history department, Tokyo Imperial Museum, the tokyo Imperial Museum, Tokyo, Japan, p. 72.; institutionCode: NSMT; collectionCode: Mo; basisOfRecord: PreservedSpecimen
- **Type status:** Other material. **Occurrence:** occurrenceDetails: https://www.gbif.org/occurrence/2864965308; catalogNumber: 2961H ex.NSMT-Mo 2961; recordNumber: 2961 in Iwakawa 1919; individualCount: 5; lifeStage: adults; preparations: shell (dried); previousIdentifications: *Viviparus sclateri* (Frauenfeld, 1865) in Tomotaro Iwakakawa. 1919. Catalogue of Japanese Mollusca in the Natural history department, Tokyo Imperial Museum, the tokyo Imperial Museum, Tokyo, Japan, p. 72.; occurrenceID: paper:SK2020:2961H; **Taxon:** scientificName: *Sinotaia quadrata histrica* (Gould, 1859); kingdom: Animalia; phylum: Mollusca; family: Viviparidae; vernacularName: Hime-tanishi (in Japanese name); **Location:** country: Japan; stateProvince: Nagano; locality: Lake Suwa; verbatimLocality: Suwako (in Japanese) [Lake Suwa]; **Identification:** identifiedBy: Takumi Saito; dateIdentified: 2020-02-03/04; **Event:** year: before 1919; **Record Level:** language: Japanese; bibliographicCitation: Tomotaro Iwakakawa. 1919. Catalogue of Japanese Mollusca in the Natural history department, Tokyo Imperial Museum, the tokyo Imperial Museum, Tokyo, Japan, p. 72.; institutionCode: NSMT; collectionCode: Mo; basisOfRecord: PreservedSpecimen

#### Diagnosis

Adult shell small and thin, but not fragile; shell shape subconical to pyramidal. Adult shell bright brown or yellowish- or reddish-brown often covered with many attachments such as alga; shell surface slightly glossy, weak growth lines and spiral lines are usually present. Suture shallow; spire rounded. Young shell small, very thin and fragile; shell shape pyramidal with rounded angle. Young shell bright olive; shell surface quite glossy with no spiral ridges on upper part of the spire.

#### Taxon discussion

*Sinotaia quadara histrica* can be distinguished from other Japanese Viviparidae by the following features, based on the illustrations and information from literature (Suppl. material 1). In particular, the adult shell width tends to be relatively smaller than that of other Japanese Viviparidae (Masuda and Uchiyama 2004; for example, under 24 mm except for one exception in Kagawa et al. (2019)).

**Figure 5.**
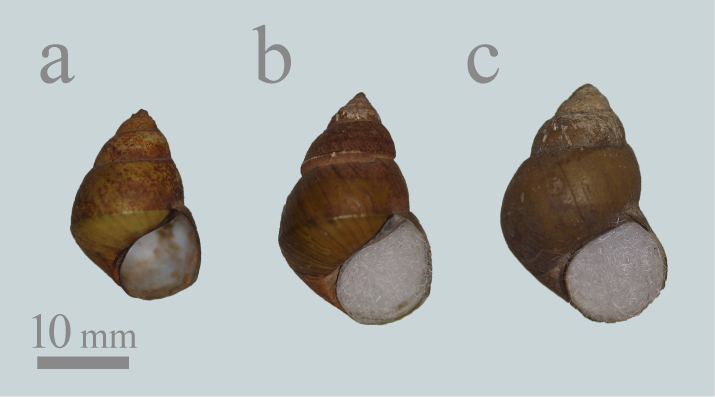
Representative shells of *Sinotaia quadrata histrica* (Gould, 1859) from Japan, deposited in the collection of the National Museum of Nature and Science, Tokyo, which were treated as *Viviparus sclateri* (Frauenfeld, 1865) in Iwakawa (1919). **a-c**: Lake Suwa, Nagano Pref. (a: 2960H, b-c: 2961H).

Firstly, *S. q. histrica* is easily distinguished from *Cipangopaludina* species/subspecies in Japan, based on the small shell width, pyramidal shell shape and the higher spire (Fig. 5).

The size of *Heterogen* sp. is larger and the apical whorls are also larger than those of *S. q. histrica.* In addition, *Heterogen* sp. has the weak spiral ridges on even the early whorls, whereas *S. q. histrica* does not have them. Diagnostic differences between *S. q. histrica* vs. *H. japonica* and *H. longispira* have been provided above, so we refer to the taxon discussions of these latter two species.

#### Notes

*Sinotaia quadara histrica* was identified in two of the 11 lots examined, which were from Lake Suwa (Fig. 5). Some specimens of *H. japonica* from Lake Suwa have a similar shell shape to *S. q. histrica*, but *S. q. histrica* has a more rounded shell apex, a broader spire angle, shallower sutures and a brighter colour than those of *H. japonica*.

We treated only the Japanese *Sinotaia* species for synonymy, as the taxonomic relationship between the continental *Sinotaia* and the Japanese *Sinotaia* species is not clear and not the point of this study. The species-group name, *nitens* was synonymised with a question mark by Pilsbry (1902) and we followed this treatment. *Vivipara lacustris* in Kawamura (1918) was documented as a small species that is distributed in the Kyushu Region. In the past, *S. q. histrica* was considered to be distributed in Kyushu Region (Pilsbry 1902) and so *V. lacustris* in Kawamura 1918 may be a junior synonym of *S. q. histrica*.

## Analysis

**Figure 6.**
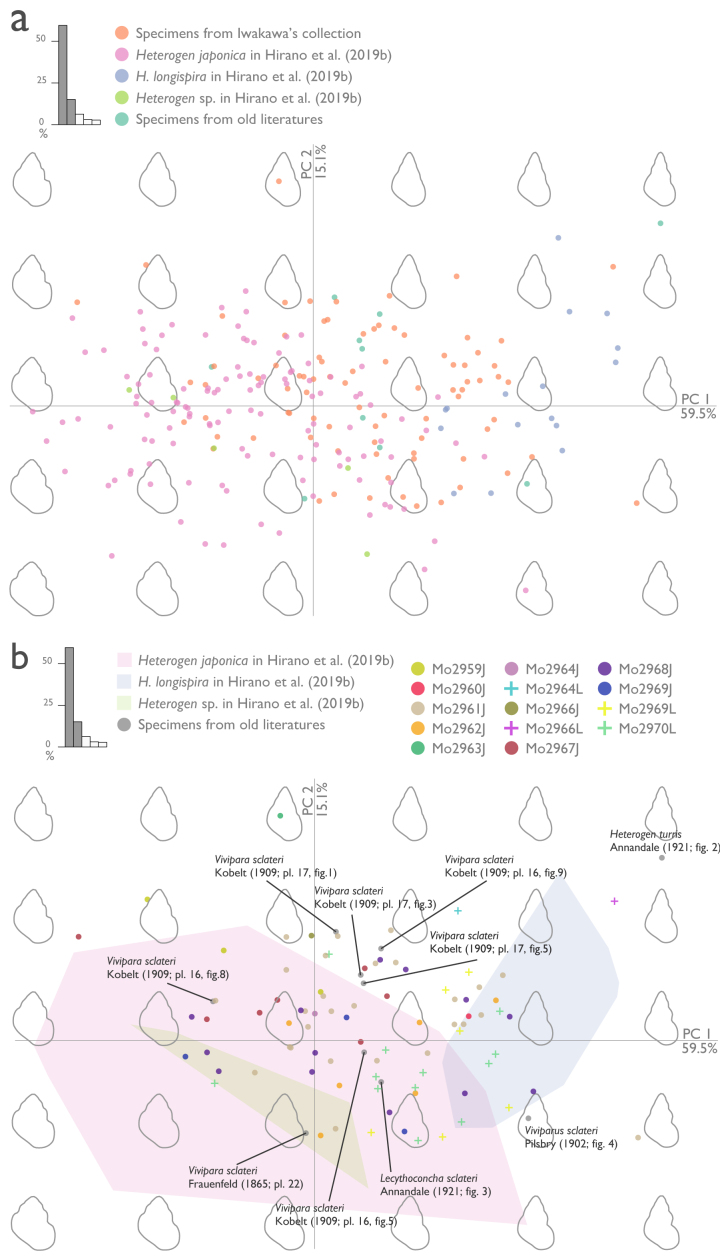
**a**: Plots of principal component analysis (PCA) of all analysed specimens. The top left graph shows the proportion of variance of each component. Figures on the background indicate reconstructed shell morphology, based on principal components. **b**: Plots of PCA of Iwakawa's specimens and old literature specimens. The morphospace occupation by specimens from Hirano et al. (2019b) is shown as coloured polygons. The top left graph shows the proportion of variance of each component. Background outlines indicate the reconstructed shell morphology, based on principal components.

**Figure 7.**
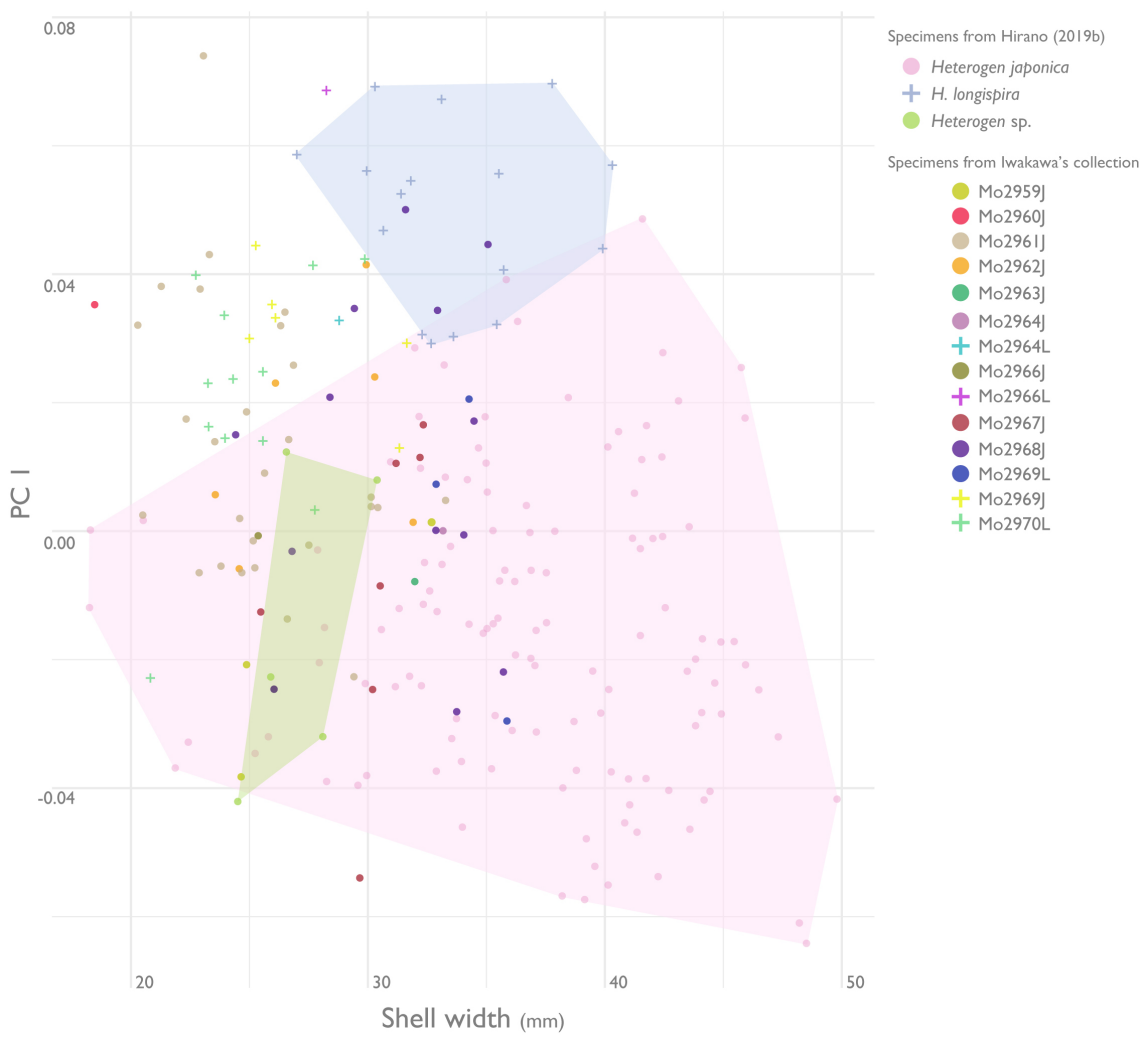
Plots of principal component axis one versus shell width for specimens from Iwakawa and Hirano et al. (2019b). The morphospace occupation by specimens from Hirano et al. (2019b) is also shown as coloured polygons.

The principal results of the PCA of EF analysis are shown in Fig. 6 and Fig. 7 (for full results of the PCA and SW, see Suppl. material 2). PC1 and PC2 explained 74.6% of the variance and the first five components explained 86.7% of the variance (PC1: 59.5%; PC2: 15.1%; PC3: 6.3%; PC4: 3.0%; PC5: 2.7%). *Heterogen japonica* and *H. longispira* from Hirano et al. (2019b) had little overlap in each region and *Heterogen* sp. was fully included in the morphological range of *H. japonica*. Many of the *H. japonica* in Iwakawa's collection identified qualitatively by diagnostic features were within the morphological range of *H. japonica* from Hirano et al. (2019b), but some were located within the range of *H. longisira* from Hirano et al. (2019b) or in parts of the morphospace not covered by either. Some of *H. longispira* in Iwakawa's collection, which were identified qualitatively, were within the morphological range of *H. longispira* from Hirano et al. (2019b), but some were located within the morphological range of *H. japonica* from Hirano et al. (2019b) or in parts of the morphospace not covered by either.

## Discussion

All eleven lots in the Iwakawa collection in the NSMT were previously thought to consist of a single species, *Viviparus sclateri* in Iwakawa (1919), but now have been found to consist of at least three species/subspecies, *H. japonica*, *H. longispira* and *Sinotaia quadrata histrica*. *H. japonica* was found in all lots studied during our investigation; *H. longispira* was found amongst four of the lots, which were all from Lake Biwa drainage (Table 1 and Fig. 1). This result reflects the taxonomic understanding of the period, prior to *H. longispira* being recognised as a distinct species (as *H. turris* in Annandale (1921)). Furthermore, all specimens of *Viviparus sclateri* in Iwakawa (1919) from outside of Lake Biwa drainage differed from *H. longispira* (Fig. 2a-h and Fig. 5), because these specimens do not have spiral ridges on the upper part of each spire which is a distinctive character of *H. longispira* (Figs 2, 4; Annandale 1921, Kihira et al. 2003). Nevertheless, almost all of these specimens seem to have a higher spire and narrower spire angle than *H. japonica* as illustrated and documented in literature (Suppl. material 1; e.g. Figure 2 in Hirano et al. 2015; Figure 1 in Hirano et al. 2019b). The higher spire and the narrower spire angle of the *H. japonica* that was listed as *Viviparus sclateri* in Iwakawa (1919) from outside of Lake Biwa basin are noteworthy. Our morphological analysis showed that some specimens of *Viviparus sclateri* in Iwakawa's collection differ in shell shape from specimens of *H. japonica* in Hirano et al. (2019b), which were collected from the entire native geographic range of the species (Fig. 6). The shell shapes of these specimens seemed to be more elongated (i.e. having the higher spire and the narrower spire angle) and some could not be distinguished from the shell shape of *H. longispira* in Hirano et al. (2019b), based on PC1 and PC2 of the EF analysis. The similarities of the shell shapes shown by the EF analysis may suggest the complexity of the taxonomic relationship between the two species. Nevertheless, most of them had the shallower suture and this seemingly differed from *H. longispira*. Furthermore, most specimens of *H. japonica* in Iwakawa's collection had a smaller shell width than *H. japonica* in Hirano et al. (2019b) (*Fig. 7*). This characteristic morphology seemed to be particularly abundant in *H. japonica* from Lake Suwa (2960J-2962J; e.g. Fig. 2c). The distinctive (see diagnosis and taxon discussion) shell morphology of *H. longispira* is considered to be a consequence of adaptation to its ancient lake habitat and this evolutionary change in morphology may have originated multiple times, based on examination of fossil specimens (Hirano et al. 2019b). Furthermore, plastic and/or adaptive morphological changes to the environment seem to occur easily both within and amongst species in viviparid gastropods (Hirano et al. 2019b, Kagawa et al. 2019, Stelbrink et al. 2020). Such morphological changes might have occurred in those *H. japonica* that have the elongated shell shape, as two of the three localities from where these specimens were collected are large lakes. In particular, Lake Suwa, where many distinctive specimens were found, was formed around 0.10 Ma (Anma et al. 1990) and *H. japonica* from Lake Suwa in Iwakawa’s collection may represent an evolutionary distinct population.

On the other hand, *Sinotaia quadrata histrica* specimens were identified only from Lake Suwa (Fig. 5). These specimens were included in *Viviparus sclateri* of Iwakawa (1919). Fig. 6 of Iwakawa (1897a) and Figs. 5-6 of Iwakawa (1897b) shown as young specimens of *Paludina ingallsiana* may also be *S. q. histrica* from Lake Suwa. The existence of *S. q. histrica* in Lake Suwa might have resulted in further taxonomic confusion. *Sinotaia q. histrica* was considered to be only distributed in the southern part of Japan during that period (Pilsbry 1902, Kuroda 1929). Perhaps, *S. q. histrica* in the Iwakawa's collection may be the oldest record of the species from the eastern part of Japan. Now, *S. q. histrica* is a common viviparid gastropod throughout Japan, except for the Ryukyu Islands (Masuda and Uchiyama 2004). The species was considered to have been introduced after the prehistoric era, based on molecular phylogenetic studies and records of shell middens' records (Kurozumi 2001, Hirano et al. 2015, Kurozumi 2019). In a recent study, further complicated history of colonisation of *S. q. histrica* was estimated by genetic markers and multiple colonisations from the continent at different times were revealed (Ye et al. 2020). The time when population established in the eastern part of Japan was estimated around 7910 years ago in this paper. However, the distribution of *S. q. histrica* has been considered to have recently expanded to the eastern part of Japan because there were few old records including from shell midden (Matsuoka 2001). Owing to thesemissing records, the history of the expansion of distribution have not been sufficiently clarified. Our probable oldest record from the eastern part of Japan may have implications for this, although further molecular and bibliographical studies are needed.

All samples from Lake Biwa drainage include *H. japonica*, though some lots contain both *H. japonica* and *H. longispira* (Table 1 and Fig. 1). The historically-documented co-existence of the two species differs from the current distribution of the two viviparid gastropods around Lake Biwa; *H. japonica* is rarely found within Lake Biwa at present (Horie 1971, Hirano et al. 2019b). Previous studies have stated that *H. longispira* is relatively common in the shallow area of southern Lake Biwa (Kuroda 1947a, Oyama and Kajiyama 1959, Kihira and Matsuda 1990). Although there is a possibility that each specimen was collected from several populations (including outside of the lake population), the co-existence of the two species in the same lot may be indicative of the past sympatric distribution of these species in Lake Biwa. Furthermore, some specimens from southern Lake Biwa and the Seta River flowing from Lake Biwa showed an intermediate morphology between *H. japonica* and *H. longispira* (Figs 3, 6; see also notes in taxon treatments of *H. longispira*). Some specimens, especially young and juvenile specimens, are easily assigned to one of the two species, based on the existence of spiral ridges and shell shapes (*H. japonica*: Fig. 3a, f and l; *H. longispira*: Fig. 3e, k and m). However, some specimens are difficult to distinguish. Namely, they have several distinctive morphological features of *H. longispira* (i.e. the pronounced spiral ridges, the shouldered whorls, the deep suture, the strong basal angle and the high spire), but the shell shapes of these specimens tend to be more similar to *H. japonica* (Fig. 6). In particular, many specimens from the Seta River have a distinctive morphology with a linear shape and smaller shell size, which is possibly indicative of a distinctive population (Figs 3, 6, 7). In addition, some specimens illustrated by past malacologists are presumed to be from southern Lake Biwa or the Seta River. For example, pl. 9, fig. 4 in Pilsbry (1902) (= pl. 16, fig. 7 in Kobelt 1909) from near Kyoto resembles specimens from the Seta River (Fig. 3i and j), which flows to Kyoto City, and pl. 10, fig. 18 in Kobelt (1879) (= pl. 16, fig. 9 in Kobelt 1909) looks similar to specimens from southern Lake Biwa (Fig. 3b). In fact, our analysis of literature illustrations and other specimens showed that the shell shapes of these specimens were similar to southern Lake Biwa or the Seta River specimens, whereas the shell shapes of illustrations of *Vivipara sclateri* from the original description were far from the shape of *H. longispira* (Fig. 6b). A molecular study using genome-wide genetic data suggested introgressive hybridisation between the two species and some genetic populations seemingly had been generated by hybridisation (Hirano et al. 2019b). As such, hybridisation may have been the cause of the formation of the populations with an intermediate morphology. To reveal the traits and origin of these unique specimens, we need to examine current specimens carefully and include molecular analyses whenever possible. However, *H. longispira* of southern Lake Biwa and the Seta River are extinct or almost extinct (Nishino 1991, Kihira et al. 2003, Kihira et al. 2009, Nakai 2016). Similarly, populations of *H. japonica* of Lake Kasumigaura and Lake Suwa are critically endangered or extinct (Kurasawa 1988, Nishiwaki et al. 2008). Examining past specimens in the Museum may be the only way to elucidate the historical distribution of these species.

## Supplementary Material

A1B13F28-AF95-5563-8478-CF0E52B5F0D6 10.3897/BDJ.8.e52233.suppl1 Supplementary material 1 Full list of synonyms and key publications of *Heterogen japonica*, *H. longispira* and *Sinotaia quadrata histrica* Data type References list Brief description The lists include synonyms and key publications of three viviparid species in Japan. File: oo_431781.docx https://binary.pensoft.net/file/431781 Takumi Saito and Osamu Kagawa

4FFA5439-E42A-51B3-BA5A-13B6F2A08D40 10.3897/BDJ.8.e52233.suppl2 Supplementary material 2 The full results of morphological analysis Data type Morphological (data) Brief description The principal component values summarised from elliptic Fourier analysis and shell width measured directly. File: oo_431796.csv https://binary.pensoft.net/file/431796 Takumi Saito and Kagawa Osamu

XML Treatment for Heterogen japonica

XML Treatment for Heterogen longispira

XML Treatment for Sinotaia quadrata histrica

## Figures and Tables

**Table 1.** List of examined viviparid lots deposited in the National Museum of Nature and Science, Tokyo, which were treated as *Viviparus sclateri* (Frauenfeld, 1865) in Iwakawa 1919. The number in each species/subspecies indicates examined individuals in each sample.

Sample No. in this study	Lot Acc. No. in the museum	Locality	*Heterogen japonica*	*Heterogen longispira*	*Sinotaia quadrata histrica*	Remarks
2959J	NSMT-Mo 2959	Japan, Ibaraki Pref., Lake Kasumigaura	3			Probably one of No. 2950 in Iwakawa (1919).
2960J	NSMT-Mo 2960	Japan, Nagano Pref., Lake Suwa	1			
2960H	NSMT-Mo 2960	Japan, Nagano Pref., Lake Suwa			1	
2961J	NSMT-Mo 2961	Japan, Nagano Pref., Lake Suwa	27			
2961H	NSMT-Mo 2961	Japan, Nagano Pref., Lake Suwa			5	
2962J	NSMT-Mo 2962	Japan, Nagano Pref., Lake Suwa	6			
2963J	NSMT-Mo 2963	Japan, The western part of Aichi Pref.	1			
2964J	NSMT-Mo 2964	Japan, Shiga Pref., Lake Biwa	1			
2964L	NSMT-Mo 2964	Japan, Shiga Pref., Lake Biwa		1		
2966J	NSMT-Mo 2966	Japan, Shiga Pref., Hikone City, Hikone	1			
2966L	NSMT-Mo 2966	Japan, Shiga Pref., Hikone City, Hikone		2		
2967J	NSMT-Mo 2967	Japan, Shiga Pref., Maibara City, Irie	7			
2968J	NSMT-Mo 2968	Japan, Shiga Pref., Tsukuma Lagoon	15			This locality was drained and converted into terrestrial areas
2969J	NSMT-Mo 2969	Japan, Shiga Pref., Otsu City, Zeze	4			
2969L	NSMT-Mo 2969	Japan, Shiga Pref., Otsu City, Zeze		6		*H. longispira* are almost extinct in this locality
2970J	NSMT-Mo 2970	Japan, Shiga Pref., the Seta River	2			
2970L	NSMT-Mo 2970	Japan, Shiga Pref., the Seta River		15		*H. longispira* are almost extinct in this locality
