# Supplementary material for: New insights from museum specimens: a case of Viviparidae (Caenogastropoda: Mollusca) in Iwakawa’s collection preserved in the National Museum of Nature and Science, Tokyo
Source: Biodivers Data J. 2020 Dec 21;8:e52233. doi: 10.3897/BDJ.8.e52233 (PMC7769901; doi:10.3897/BDJ.8.e52233)
Supplement: Supplementary material 1 — Full list of synonyms and key publications of Heterogen japonica, H. longispira and Sinotaia quadrata histrica [file bdj-08-e52233-s001.docx]

**Supplementary file 1**

Full list of synonyms and key publications of *Heterogen japonica*, *H. longispira*, and *Sinotaia quadrata histrica*

Supplement of New insights from museum specimens: a case of Viviparidae (Caenogastropoda: Mollusca) in Iwakawa’s collection preserved in the National Museum of Nature and Science, Tokyo

by Takumi Saito and Osamu Kagawa

***Heterogen japonica* (Martens, 1861)**

- *Paludina japonica* von Martens 1861: 44. Type locality: Japan. (original description); Reeve 1862: pl. 3, figs 13a–b; Kobelt 1879: 120, pl. 11, fig. 1; Iwakawa 1895: 356, pl. 19, fig. 1; Tanba 1897: 431.
- *Vivipara sclateri* Frauenfield 1865: 531, pl. 22, unnumbered figs. Type locality: Japan. (original description); Pilsbry 1895: 158; Kobelt 1909: 102, pl. 16, figs 3–6, 8 [part; pl. 16, fig. 9 and pl. 17, figs 3, 5 seemingly have the intermediate morphology between *H. japonica* and *H. longispira*, see discussion]; Annandale 1916: 46 [part].
- *Paludina ingallsiana* — Kobelt 1879: 124, pl. 10, fig. 14 [part; pl. 10, figs 15–16 and 18 seemingly have the intermediate morphology between *H. japonica* and *H. longispira*, see discussion]; Iwakawa 1895: 412, pl. 19, figs 8–9 [part]; Iwakawa 1897a: 86, pl. 5, fig. 7 [part] (Japanese viviparid catalogue); Iwakawa 1897b: 5.
- *Paludina oxytropis* — Kobelt 1879: 123, pl. 11, fig. 6; Iwakawa 1895: 411, pl. 19, figs 5–7; Iwakawa 1897a: 9, pl. 2, fig. 10; Iwakawa 1897b: 88, pl. 5, figs 8–12.
- *Paludina sclateri* — Kobelt 1879: 121, pl. 11, fig. 3; Tanba et al. 1883: 368; Tanba et al. 1891: 216; Iwakawa 1895: 357, pl. 19, fig. 2; Kitahara 1895: 89.
- *Paludina oxytropis* var. *japonica* — Iwakawa 1897a: 88, pl. 5, figs 15, 17 [Iwakawa noted that fig. 15 was intermediate form between *P. oxytropis oxytropis* and *P. o. japonica*]; Iwakawa 1897b: 9, pl. 2, fig. 13.
- *Paludina oxytropis* var. *sclateri* — Iwakawa 1897a: 89, pl. 5, figs 13–14, 16 [Iwakawa noted that figs 13, 16 were intermediate form between *P. oxytropis oxytropis* and *P. o. sclateri*]; Iwakawa 1897b: 9, pl. 2, figs 7–9, 11.
- *Vivipara oxytropis* — Pilsbry 1895: 158.
- *Viviparus japonicus* — Pilsbry 1902: 117, pl. 9, fig. 1; Hirase 1909: 45; Hirase 1910: 15; Hatta and Sasaki 1910: 96; Lake Biwa fisheries experimental station 1915: 30, fig. 12; Yagura 1916: 18; Iwakawa 1919: 70–71; Yamakawa 1930: pl. 48, fig. 11; Hirase 1934a: 55; Hirase 1934b: pl. 77, fig. 9; Yagura 1935: 12; Hirase and Taki 1951: pl. 77, fig. 9; Hirase and Taki 1954: pl. 77, fig. 9; Taki 1954: 186, fig. 340.
- *Viviparus japonicus* var. *iwakawa* Pilsbry 1902: pl. 9, fig. 3. Type locality: Furukawa, Rikuzen [Furukawa City, Miyagi Pref., Japan]. (original description).
- *Viviparus sclateri* — Pilsbry 1902: 118 [part]; Iwakawa 1919: 72 [part].
- *Vivipara japonica* — Kobelt 1909: 99, pl. 15, figs 1–4; Hannibal 1911: 32; Annandale 1916: 46; Kawamura 1918: 358.
- *Vivipara japonica* var. *iwakawa* — Kobelt 1909: 100, pl. 15, figs 5–7.
- *Idiopoma* (*Idiopoma*) *japonica* — Hannibal 1912: 194.
- *Viviparus japonicus iwakawa* — Iwakawa 1919: 71–72; Hirase 1934a: 55; Hirase 1934b: pl. 77, fig. 10; Yagura 1935: 13; Yamakawa 1953: pl. 46, fig. 3; Hirase and Taki 1951: pl. 77, fig. 10; Hirase and Taki 1954: pl. 77, fig. 10.
- *Viviparus japonecus* — Kanamaru 1920: 4, pl. 2, fig. 85. [sic].
- *Lecythoconcha japonica* — Annandale 1921: 401; Annandale 1922: 133.
- *Lecythoconcha sclateri* — Annandale 1921: 401, fig.3; Annandale 1922: 133.
- *Viviparus* (*Idiopoma*) *japonicus* — Hirase 1927: 1380, fig. 2653.
- *Viviparus* (*Idiopoma*) *japonicus iwakawa* — Hirase 1927: 1381, fig. 2654.
- *Vivipara japonica* var. *iwakawae* — Prashad 1928: 173, pl. 19, fig. 12. [sic].
- *Viviparus* (*Cipangopaludina*) *japonicus* — Prashad 1928: 172; Kuroda 1929: 102; Kuroda 1933: 182; Kuroda 1935: 44; Taki, 1933 in Horikoshi and Itabashi 1994: 20; Yagura 1932: 28; Yagura 1938: 16; Taki 1938: 13; Nishimura and Watabe 1943: 65; Okada and Kurasawa 1950: 151, figs 5–6, 9, 14 (in text), pl. 2, figs 5–6, pl. 4, figs a–g.
- *Viviparus* (*Dactylochlamys*) *iwakawae* — Prashad 1928: 173, pl. 19, fig. 12. [sic].
- *Viviparus* (*Viviparus*) *sclateri* — Prashad 1928: 172, pl. 19, fig. 5 [part?].
- *Viviparus* (*Cipangopaludina*) *japonicus iwakawa* — Kuroda 1929: 102; Yagura 1938: 16; Okada and Kurasawa 1950: 151, figs 7–9, 14 (in text), pl. 2, figs 7–8, pl. 4, figs a'–g'.
- *Viviparus* (*Cipangopaludina*) *japonicus sclateri* — Kuroda 1929: 102; Yagura 1932: 28.
- *Viviparus oxytropis* — Taki, 1933 in Horikoshi and Itabashi 1994: 20.
- *Viviparus iwakawai* — Taki 1946: 202, fig. 8 [sic].
- *Cipangopaludina japonica* — Hirase and Kuroda 1947a: 1161, fig. 3304; Kuroda 1947: 3; Kuroda 1948: 26; Kuroda 1955: 2; Kuroda 1963: 14; Kira 1959: 170, pl. 63, fig. 8; Oyama and Kajiyama 1959: 119, text-figs; Taki 1960: 170, pl. 80, fig. 12; Kuroda and Habe 1965a: 48, fig. 149; Habe and Kosuge 1967: 27, pl. 11, figs 3–5; Mori 1971: 302, fig. 15; Habe 1973: 313, pl. 17-1, fig. 28; Habe 1975: 63, 179, unnumbered figs; Kihira and Matsuda 1990: 29, text-figs; Nishino 1991: 3, text-figs; Kihira et al. 2003: 37, text-figs; Kihira et al. 2009: 37, text-figs; Masuda and Uchiyama 2004: 52, text-figs; Matsuda and Nakai 2006a: 499; Hirano et al. 2015: 435, figs 2–3; Nakai 2016a: 587; Van Bocxlaer and Strong 2016:
- *Cipangopaludina japonica iwakawa* — Hirase and Kuroda 1947b: 1162, fig. 3305; Habe and Kosuge 1967: 27.
- *Cipangopaludina* (*Ussuriensis*?) *japonica* — Habe 1990: 4.
- *Heterogen japonica* — Hirano et al. 2019b: 5033, figs 1, 4–5, 8.

***Heterogen longispira* (Smith, 1886)**

- *Paludina ingallsiana*— Kobelt 1879: 124, pl. 10, figs 15–18, pl. 11, fig. 2 [part; pl. 10, figs 15–16 and 18 seemingly have the intermediate morphology between *H. japonica* and *H. longispira*, see discussion]; Tanba et al. 1883: 368; Iwakawa 1895: 412 [part]; Iwakawa 1897a: 86 [part]; Iwakawa 1897b: 5 [part].
- *Paludina longispira*Smith 1886: 57–58. Type locality: Lake Biwa. (original description).
- *Viviparus sclateri*— Pilsbry 1902: 118, pl.9, fig. 4 [part; see discussion]; Hirase 1909: 45 [part?]; Hirase 1910: 15 [part?]; Lake Biwa fisheries experimental station 1915: 30, fig. 13; Iwakawa 1919: 72 [part].
- *Vivipara sclateri*— Kobelt 1909: 102, pl. 16, figs 7, 9, pl. 17, figs 1–5. [part; pl. 16, fig. 9 and pl. 17, figs 3, 5 seemingly have the intermediate morphology between *H. japonica* and *H. longispira*, see discussion]; Annandale 1916: 46 [part]; Kawamura 1918: 358, fig. 441 [part?].
- *Heterogen turris* Annandale 1921: 400, figs 1–2. Type locality: Lake Biwa. (original description); Annandale 1922: 133; Oyama and Kajiyama 1959: 119, text-figs.
- *Viviparus* (*Heterogen*) *turris*— Hirase 1927: 1381, fig. 2655.
- *Viviparus* (*Heterogen*) *longispira*— Prashad 1928: 172, pl. 19, fig. 7; Kuroda 1929: 102; Hirase 1934b: pl. 77, fig. 12; Yagura 1935: 13; Yagura 1938: 16; Hirase and Kuroda 1947c: 1161, fig. 3302.
- *Heterogen longispira*— Kuroda 1947: 3; Kuroda 1948: 26; Kuroda 1955: 2; Kira 1959: 170, pl. 63, fig. 7; Taki 1960: 170, pl. 80, fig. 13; Kuroda 1963: 14; Mori 1971: 302, fig. 11; Habe 1973: 313, pl. 17-1, fig. 27; Habe 1975: 63, 243, unnumbered figs; Habe 1990: 4; Kihira and Matsuda 1990: 32, text-figs; Nishino 1991: 4, text-figs; Kihira et al. 2003: 39, text-figs; Kihira et al. 2009: 39, text-figs; Matsuda and Nakai 2006b: 495, unnumbered fig.; Hirano et al. 2015: 435, figs 2–3; Hirano et al. 2019b: 5033, figs 1, 4–5, 8; Nakai 2016b: 583, unnumbered fig.
- *Viviparus* (*Heterogen*) *turis* — Okada and Kurasawa 1950: 153, fig. 10–11, 14, (in text), pl. 2, figs 7–8, pl. 3, figs 9–10, pl. 4, figs a"–g". [sic].
- *Viviparus longispira* — Hirase and Taki 1951: pl. 77, fig. 10; Hirase and Taki 1954: pl. 77, fig. 12; Yamakawa 1953: pl. 46, fig. 1.
- *Cipangopaludina* (*Heterogen*) *longispira* — Kuroda and Habe 1965b: 48, fig. 151; Habe and Kosuge 1967: 27, pl. 11, figs 1–2.

***Sinotaia quadrata histrica* (Gould, 1859)**

- *Paludina histrica*Gould 1859: 41. Type locality: Ousima and Loo Choo [Amami-Oshima and the Ryukyu Islands]. (original description); Gould 1862: 106; Johnson 1964: 88, pl. 43, fig. 5 (Lectotype was selected. Type locality: "presumably" Amami-O-shima).
- [?]*Paludina nitens*Reeve 1863: pl. 10, fig. 59. Type locality: Japan. (original description); Kobelt 1879: 125; Iwakawa 1895: 413.
- *Paludina ingallsiana*— Iwakawa 1897a: 86, pl. 5, figs 5–6 [part]; Iwakawa 1897b: 5, pl. 2, fig. 6 [part].
- *Viviparus histricus*— Pilsbry 1902: pl. 9, fig. 5; Kuroda 1935: 43.
- *Vivipara histrica*— Kobelt 1909: 107, pl. 16, fig. 6.
- [?]*Vivipara nitens*— Kobelt 1909: 107.
- [?]*Vivipara lacustris*— Kawamura 1918: 358.
- *Viviparus quadratus*var.? *histricus*— Hirase 1927: 1382, fig. 2656.
- *Cipangopaludina histrica*— Kuroda 1928: 32.
- *Viviparus* (*Viviparus*) *histricus*— Prashad 1928: 172; Kuroda 1929: 102; Yagura 1938: 16.
- *Viviparus histricus*— Iwakawa 1919: 72; Yamakawa 1930: pl. 48, fig. 10; Hirase 1934a: 55; Hirase 1934b: pl. 77, fig. 11; Hirase and Taki 1951: pl. 77, fig. 11; Hirase and Taki 1954: pl. 77, fig. 11.
- *Viviparus* (*Sinotaia*) *histricus*— Hirase and Kuroda 1947d: 1160, fig. 3301.
- *Taia* (*Sinotaia*) *histrica*— Kuroda 1947: 4; Taki 1960: 170, pl. 80, fig. 14.
- *Sinotaia histrica*— Kuroda 1948: 26; Kuroda 1955: 2; Kuroda 1963: 14; Kira 1959: 169, pl. 63, fig. 5; Oyama and Kajiyama 1959: 119; Mori, 1971: 302.
- *Viviparus* (*Idiopoma*) *histricus*— Okada and Kurasawa 1950: 149, figs 3–4, 12–14 (in text), pl. 1, figs 1–2, pl. 3, figs a'–g'.
- *Sinotaia quadratus histrica*— Kuroda and Habe 1965c: 48, fig. 152.
- *Sinotaia quadrata histrica*— Habe and Kosuge 1967: 27, pl. 11, fig. 8; Habe 1973: 313, pl. 17-1, fig. 26; Habe 1975: 15, 63, 259, unnumbered figs; Habe 1990: 4; Kihira and Matsuda 1990: 34, text-figs; Nishino 1991: 5, text-figs; Kihira et al. 2003: 41, text-figs; Kihira et al. 2009: 41, text-figs; Masuda and Uchiyama 2004: 54, text-figs; Hirano et al. 2015: 435, figs 2–3.

**References**

Annandale N (1916) Zoological results of a tour in the Far East. The Mollusca of Lake Biwa, Japan. Memoirs of the Asiatic Society of Bengal 6 (1): 39‐74. URL: https://biodiversitylibrary.org/page/47681217

Annandale N (1921) Zoological results of a tour in the Far East. The viviparous water-snail of Lake Biwa, Japan. Memoirs of the Asiatic Society of Bengal 6 (7): 397‐401. URL: https://biodiversitylibrary.org/page/47681678

Annandale N (1922) The macroscopic fauna of Lake Biwa. Annotationes Zoologicae Japonenses 10: 127‐153.

Frauenfeld GRV (1865) Zoologische Miscellen. IV. Verhandlungen der Kaiserlich-Königlichen Zoologisch-Botanischen Gesellschaft in Wien 15: 259‐266. URL: https://www.biodiversitylibrary.org/page/16400700

Gould AA (1859) Dr. A. A. Gould read the following descriptions of new species of shells. Proceedings of the Boston Society of Natural History 7: 40‐45. URL: https://www.biodiversitylibrary.org/page/9249577

Gould AA (1862) Otia conchologica: descriptions of shells and mollusks, from 1839 to 1862. Gould and Lincoln, Boston, 256 pp. https://doi.org/10.5962/bhl.title.10460

Habe T (1973) Mollusca. In: Ueno M (Ed.) The late Tamiji Kawamura freshwater biology of Japan, enlarged and revised edition. Hokuryu-kan, Tokyo, 309-341 pp. [In Japanese with English title].

Habe T (1975) A illustrated handbook for school student by Gakken, 7. Mollusca, I. Gastropods. Gakusyu-kenkyu-sya, Tokyo, 301 pp. [In Japanese].

Habe T (1990) The list of Japanese freshwater mollusks: 1. Hitachiobi 54: 3‐6. [In Japanese].

Habe T, Kosuge S (1967) A standard illustrated handbook in natural color. III. Mollusca. Hoiku-sya, Osaka, 223 pp. [In Japanese].

Hannibal H (1911) Further notes on Asiatic Viviparas in California. The Nautilus 25 (2): 31‐32. URL: https://www.biodiversitylibrary.org/page/26379826

Hannibal H (1912) A synopsis of the recent and tertiary freshwater Mollusca of the Californian province, based upon an ontogenetic classification. Proceedings of the Malacological Society of London 10: 112‐211. URL: https://www.biodiversitylibrary.org/page/15237075

Hatta S, Sasaki N (1910) A list of the gastropods and lamellibranchs of Hokkaido. Transactions of the Sapporo Natural History Society 3: 93‐98. [In Japanese with English title and abstract]. URL: http://hdl.handle.net/2115/60834

Hirano T, Saito T, Chiba S (2015) Phylogeny of freshwater viviparid snails in Japan. Journal of Molluscan Studies 81 (4): 435‐441. https://doi.org/10.1093/mollus/eyv019

Hirano T, Saito T, Tsunamoto Y, Koseki J, Prozorova L, Do VT, Matsuoka K, Nakai K, Suyama Y, Chiba S (2019b) Role of ancient lakes in genetic and phenotypic diversification of freshwater snails. Molecular Ecology 28 (23): 5032‐5051. https://doi.org/10.1111/mec.15272

Hirase S (1927) Mollusca. In: Uchida S (Ed.) An illustrated encyclopedia of the fauna of Japan. Hokuryu-kan, Tokyo. [In Japanese].

Hirase S (1934a) Mollusca. In: Gunma Prefecture (Ed.) The catalogue of Imperial inspection. III. Peculiar natural history specimens. Gunma Prefecture, Maebashi, 35-79 pp. [In Japanese].

Hirase S (1934b) A collection of Japanese shells with illustration in natural colours. Matsumura- sansho-do, Tokyo, 129 pls and 88 pp. [In Japanese with English titile].

Hirase S, Kuroda T (1947a) *Cipangopaludina japonica* (Martens). In: Uchida S (Ed.) An illustrated encyclopedia of the fauna of Japan, revised ver. Hokuryu-kan, Tokyo, 1161-1161 pp. [In Japanese].

Hirase S, Kuroda T (1947b) *Cipangopaludina japonica iwakawa* (Pilsbry). In: Uchida S (Ed.) An illustrated encyclopedia of the fauna of Japan, revised ver. Hokuryu-kan, Tokyo, 1162-1162 pp. [In Japanese].

Hirase S, Kuroda T (1947c) *Viviparus* (*Heterogen*) *longispira* (Smith). In: Uchida S (Ed.) An illustrated encyclopedia of the fauna of Japan, revised ver. Hokuryu-kan, Tokyo, 1161-1161 pp. [In Japanese].

Hirase S, Kuroda T (1947d) *Viviparus* (*Sinotaia*) *histricus* (Gould). In: Uchida S (Ed.) An illustrated encyclopedia of the fauna of Japan, revised ver. Hokuryu-kan, Tokyo, 1160-1160 pp. [In Japanese].

Hirase S, Taki Is (1951) A handbook of illustrated shells in natural colors from Japanese Islands and their adjacent territories. Bunkyo-kaku, Tokyo, 134 pls and 43 pp. [In Japanese with English title].

Hirase S, Taki Is (1954) An illustrated handbook of shells in natural colors from Japanese Islands and their adjacent territories. Maruzen, Tokyo, pls. 124 and 10 pp. [In Japanese with English title].

Hirase Y (1909) Introduction book of Mollusks. Hirase-kaikan, Kyoto, 112 pp. [In Japanese]. URL: https://dl.ndl.go.jp/info:ndljp/pid/993608

Hirase Y (1910) One thousand kinds of shells existing in Japan. Hirase-kaikan, Kyoto, 48 pp. [In Japanese]. URL: https://dl.ndl.go.jp/info:ndljp/pid/994074

Horikoshi M, Itabashi Y (1994) Republication of "List of molluscs collected from Yokohama and its environs" compiled by the late Dr. Isao Taki in 1933 part 1. Natural History Report of Kanagawa (16): 17‐28. URL: http://nh.kanagawa- museum.jp/files/data/pdf/nhr/16/nhr16_017_028horikoshi.pdf

Iwakawa T (1895) Freshwater mollusks in Japan (2). Zoological magazine 7 (86): 411‐414. [In Japanese]. URL: https://dl.ndl.go.jp/view/download/digidepo_10825080_po_ART0003837109.pdf? contentNo=1&alternativeNo=

Iwakawa T (1897a) Freshwater mollusks in Japan (2), with plate 2. Zoological Magazine 9 (99): 5‐10. [In Japanese]. URL: https://dl.ndl.go.jp/view/download/digidepo_10825396_po_ART0003840054.pdf? contentNo=1&alternativeNo=

Iwakawa T (1897b) Notes on the *Paludina*-species of Japan. Annotationes Zoologicae Japonenses 1: 83‐92. URL: https://dl.ndl.go.jp/view/download/digidepo_10852805_po_ART0003842255.pdf? contentNo=1&alternativeNo=

Iwakawa T (1919) Catalogue of Japanese Mollusca in the Natural history department, Tokyo Imperial Museum. Tokyo Imperial Museum, Tokyo, 375 pp. [In Japanese].

Kanamaru T (1920) An investigation report of Mollusca in Mie Prefecture. Mie Prefecture, Tsu, 17 pp. [In Japanese].

Kawamura T (1918) Japanese freshwater biology. I. Shoka-bo, Tokyo, 362 pp. [In Japanese]. https://doi.org/10.11501/956620

Kihira H, Matsuda M (1990) Freshwater mollusks in Lake Biwa and the Yodo River. Tatara-syobo, Hirakata, 131 pp. [In Japanese].

Kihira H, Matsuda M, Uchiyama R (2003) Freshwater Molluscs of Japan 1: Freshwater Molluscs of Lake Biwa and the Yodo River. Pisces, Tokyo, 159 pp. [In Japanese].

Kihira H, Matsuda M, Uchiyama R (2009) Freshwater Molluscs of Japan 1: Freshwater Molluscs of Lake Biwa and the Yodo River, revised ver. Pisces, Tokyo, 159 pp. [In Japanese].

Kira T (1959) An illustrated book of Japanese mollusks in natural colors, revised ver. Hoiku-sya, Osaka, 239 pp. [In Japanese].

Kitahara T (1895) Animals in Lake Kasumigaura. Zoological Magazine 7 (77): 87‐90. [In Japanese]. URL: https://dl.ndl.go.jp/view/download/digidepo_10824909_po_ART0003801509.pdf? contentNo=1&alternativeNo=

Kobelt W (1879) Fauna molluscorum extramarinorum Japoniae, nach den von Professor Rein gemachten Sammlungen. Christian Winter, Frankfurt a. M., 171 pp. https://doi.org/10.5962/bhl.title.12881

Kobelt W (1909) Die Gattung *Paludina* Lam. (Vivipara Montfort): neue Folge. In: Martini FHW, Chemnitz JH (Eds) Sytematisches Conchylien-Cabinet. Bd. 1 Abt. 21a. Verlag von Bauer und Raspe, Nürnberg, 98-430 pp. https://doi.org/10.5962/bhl.title.119758

Kuroda T (1928) A catalogue of mollusks in Amami-Oshima. Kagoshima Prefecture, Kagoshima, 120 pp. [In Japanese].

Kuroda T (1929) Notes on the nomenclature of the Japanese species of the genus *Viviparus*. Venus 1 (3): 98‐102. [In Japanese]. https://doi.org/10.18941/venusomsj.1.3_98

Kuroda T (1933) A catalogue of Mollusca in Fukui Prefecture. In: Fukui Prefecture (Ed.) A catalogue of living things in Fukui Prefecture. Fukui Prefecture, Fukui, 169-205 pp. [In Japanese].

Kuroda T (1935) A catalogue of Mollusca in Miyazaki Prefecture. Miyazaki Prefecture, Miyazaki, 71 pp. [In Japanese].

Kuroda T (1947) Classified list of freshwater shells from the Japanese Islands. Mokuhachi-tengu- sya, Kyoto, 22 pp. [In Japanese with English title].

Kuroda T (1948) A list of mollusks in Lake Biwa. Yume-hamaguri (22): 26‐27. [In Japanese].

Kuroda T (1955) A list of freshwater molluscan shells of Japan. Yume-hamaguri (80) (Supplement): 1‐12. [In Japanese with English title].

Kuroda T (1963) A catalogue of the non-marine mollusks of Japan, including the Okinawa and Ogasawara Islands. The Malacological Society of Japan, Tokyo, 70 pp. [In Japanese with English title].

Kuroda T, Habe T (1965a) *Cipangopaludina japonica* (v. MARTENS). In: Okada Y, Uchida S, Uchida T (Eds) New illustrated encyclopedia of the fauna of Japan (vol. 2). Hokuryu-kan, Tokyo, 48 pp. [In Japanese with English title].

Kuroda T, Habe T (1965b) *Cipangopaludina* (*Heterogen*) *longispira* (SMITH). In: Okada Y, Uchida S, Uchida T (Eds) New illustrated encyclopedia of the fauna of Japan (vol. 2). Hokuryu-kan, Tokyo, 48 pp. [In Japanese with English title].

Kuroda T, Habe T (1965c) *Sinotaia quadratus histrica* (GOULD). In: Okada Y, Uchida S, Uchida T (Eds) New illustrated encyclopedia of the fauna of Japan (vol. 2). Hokuryu-kan, Tokyo, 48 pp. [In Japanese with English title].

Lake Biwa fisheries experimental station (1915) Fisheries research report of Lake Biwa (2): Mollusks of Lake Biwa. Lake Biwa fisheries experimental station, Fukuman [Hikone], 36 pp. [In Japanese]. https://doi.org/10.11501/928661

Masuda O, Uchiyama R (2004) Freshwater mollusks of Japan 2: freshwater mollusks of Japan, including brackish water species. Pisces, Tokyo, 240 pp. [In Japanese].

Matsuda M, Nakai K (2006a) *Cipangopaludina japonica*. In: Scientific Committee for Research into the Wildlife in Shiga Prefecture (Ed.) Red data book of Shiga Prefecture 2005. Sunrise Publishing, Hikone, 499-499 pp. [In Japanese].

Matsuda M, Nakai K (2006b) *Heterogen longispira*. In: Scientific Committee for Research into the Wildlife in Shiga Prefecture (Ed.) Red data book of Shiga Prefecture 2005. Sunrise Publishing, Hikone, 495-495 pp. [In Japanese].

Mori S (1971) Mollusks in Lake Biwa. In: The scientific investigation committee of Lake Biwa Quasi-national park (Ed.) The scientific report of Lake Biwa Quasi-national park. The scientific investigation committee of Lake Biwa Quasi-national park, Otsu, 301-312 pp. [In Japanese].

Nakai K (2016a) *Cipangopaludina japonica*. In: Scientific Committee for Research into the Wildlife in Shiga Prefecture (Ed.) Red data book of Shiga Prefecture 2015. Sunrise Publishing, Hikone, 587- 587 pp. [In Japanese].

Nakai K (2016b) *Heterogen longispira*. In: Scientific Committee for Research into the Wildlife in Shiga Prefecture (Ed.) Red data book of Shiga Prefecture 2015. Sunrise Publishing, Hikone, 583- 583 pp. [In Japanese].

Nishimura T, Watabe K (1943) A checklist of mollusks in Oga Peninsula. Miscellaneous reports of Research Institute for Natural Resources 3: 63‐74. [In Japanese].

Nishino M (1991) Benthos in Lake Biwa —Wildlife of the shores. Mollusca. Lake Biwa Research Institute, Shiga Prefecture, Otsu, 46 pp. [In Japanese].

Okada Y, Kurasawa H (1950) A study of Viviparidae in Japan. In: Okada Y (Ed.) Studies of fishery animals (1). Japan publishing cooperation, Tokyo, 141-165 pp. [In Japanese]. https://doi.org/10.11501/2470294

Oyama K, Kajiyama H (1959) The relationship between *Heterogen turris* and *Cipangopaludina japonica* (a preliminary note). Yume-hamaguri (98): 1‐4. [In Japanese].

Pilsbry HA (1895) Appendix I. List of land and freshwater mollusks collected in Japan by Frederick Stearns. In: Pilsbry HA (Ed.) Catalogue of the marine mollusks of Japan, with descriptions of new species and notes on others collected by Frederick Stearns. Frederick Stearn, Detroit, 155-159 pp. https://doi.org/10.5962/bhl.title.32672

Pilsbry HA (1902) Revision of Japanese Viviparidae, with notes on *Melania* and *Bithynia*. Proceedings of the Academy of Natural Sciences of Philadelphia 54 (1): 115‐121. URL: https://www.jstor.org/stable/4062827

Prashad B (1928) Recent and fossil Viviparidae. A study in distribution, evolution, paleogeography. Memoirs of the Indian Museum 8 (4): 153‐253.

Reeve LA (1862) Monograph of the genus *Paludina*. Pl. III. In: Reeve LA (Ed.) Conchologia Iconica: or, Illustrations of the Shells of Molluscous Animals. 14. Lovell Reeve, London. URL: https://www.biodiversitylibrary.org/page/10971836

Reeve LA (1863) Monograph of the genus *Paludina*. Pl. X. In: Reeve LA (Ed.) Conchologia Iconica: or, Illustrations of the Shells of Molluscous Animals. 14. Lovell Reeve, London. URL: https://www.biodiversitylibrary.org/page/1097186

Smith EA (1886) Descriptions of three new species of freshwater shells from Japan. Journal of Conchology 5: 57‐59. URL: https://biodiversitylibrary.org/page/31653661

Taki Is (1946) The freshwater mollusks in Musashino. Collecting and breeding 8 (11): 202. [In Japanese].

Taki Is (1954) Mollusca. In: Okada Y, Taki Is, Sakai T, Abe T (Eds) An illustrated handbook of aminals in natural colors for student II. Aquatic animals. Hokyuryu-kan, Tokyo, 293-392 pp. [In Japanese]. https://doi.org/10.11501/1370534

Taki Iw (1938) A catalogue of Mollusca in Hiroshima Prefecture. Hiroshima Prefecture, Hiroshima, 33 pp. [In Japanese].

Taki Iw (1960) Gastropoda. In: Okada Y, Taki Iw (Eds) Encyclopaedia zoologica illustrated in colours vol. III. Hokuryu-kan, Tokyo. [In Japanese with English title].

Tanba K (1897) An investigation of Animals distribution in the part of Mie Prefecture. Zoological Magazine 9 (109): 428‐432. [In Japanese]. URL: https://dl.ndl.go.jp/view/download/digidepo_10825619_po_ART0003789897.pdf? contentNo=1&alternativeNo=

Tanba K, Shibata S, Takamatsu K (1883) General Zoology. Shimamura Toshisuke, Maruya-zenshiti, and Tyu-kin-do, Tokyo, 397 pp. [In Japanese]. https://doi.org/10.11501/832886

Tanba K, Shibata S, Takamatsu K (1891) General Zoology, revised ver. Shimamura Toshisuke, Maruzen, Nanko-do, and Umehara Kameshichi, Tokyo, 336 pp. [In Japanese]. https://doi.org/10.11501/832887

Van Bocxlaer B, Strong E (2016) Anatomy, functional morphology, evolutionary ecology and systematics of the invasive gastropod Cipangopaludina japonica (Viviparidae: Bellamyinae). Contributions to Zoology 85 (2): 235‐263. https://doi.org/10.1163/18759866-08502005

von Martens E (1861) Die Japanesischen Binnenschnecken im Leidner Museum. Malakozoologische Blätter 7: 32‐61. URL: https://biodiversitylibrary.org/page/15919572

Yagura H (1935) A research of viviparid gastropods and land snails. Bulletin of the Society for Hyogo Natural History (9): 7‐13. [In Japanese].

Yagura W (1916) A catalogue of Mollusca in Hyogo. Maiko-kai-kan, Tarumi [Kobe], 87 pp. [In Japanese]. https://doi.org/10.11501/954700

Yagura W (1932) A catalogue of Mollusca in Hyogo, new and revised ver. Konan-Kairui-so, Nishinomiya. [In Japanese]. https://doi.org/10.11501/1903884

Yagura W (1938) A catalogue of Japanese land mollusks (4), freshwater mollusks and harf-brackish water mollusks. Bulletin of the Society for Hyogo Natural History (15): 15‐25. [In Japanese].

Yamakawa S (1930) A illustration of mollusks in natural color. Sansei-do, Tokyo, 48 pls. and 15 pp. [In Japanese]. https://doi.org/10.11501/1173894

Yamakawa S (1953) A new illustrated handbook of shells in natural colors. Kazama-syobo, Tokyo, 60 pls. and 20 pp. [In Japanese].
